# Stabilizing non-iridium active sites by non-stoichiometric oxide for acidic water oxidation at high current density

**DOI:** 10.1038/s41467-023-43466-x

**Published:** 2023-11-23

**Authors:** Lingxi Zhou, Yangfan Shao, Fang Yin, Jia Li, Feiyu Kang, Ruitao Lv

**Affiliations:** 1 State Key Laboratory of New Ceramics and Fine Processing, School of Materials Science and Engineering, Tsinghua University, Beijing, 100084 China; 2 Institute of Materials Research and Shenzhen Geim Graphene Center, Tsinghua Shenzhen International Graduate School, Tsinghua University, Shenzhen, 518055 China; 3 Key Laboratory of Advanced Materials (MOE), School of Materials Science and Engineering, Tsinghua University, Beijing, 100084 China

**Keywords:** Electrocatalysis, Electrocatalysis

## Abstract

Stabilizing active sites of non-iridium-based oxygen evolution reaction (OER) electrocatalysts is crucial, but remains a big challenge for hydrogen production by acidic water splitting. Here, we report that non-stoichiometric Ti oxides (TiO_x_) can safeguard the Ru sites through structural-confinement and charge-redistribution, thereby extending the catalyst lifetime in acid by 10 orders of magnitude longer compared to that of the stoichiometric one (Ru/TiO_2_). By exploiting the redox interaction-engaged strategy, the in situ growth of TiO_x_ on Ti foam and the loading of Ru nanoparticles are realized in one step. The as-synthesized binder-free Ru/TiO_x_ catalyst exhibits low OER overpotentials of 174 and 265 mV at 10 and 500 mA cm^−2^, respectively. Experimental characterizations and theoretical calculations confirm that TiO_x_ stabilizes the Ru active center, enabling operation at 10 mA cm^−2^ for over 37 days. This work opens an avenue of using non-stoichiometric compounds as stable and active materials for energy technologies.

## Abstract (web summary)

Developing stable and efficient non-iridium-based acidic oxygen evolution reaction electrocatalysts is crucial but challenging. Here, we report that non-stoichiometric Ti oxides can safeguard the Ru sites through charge-redistribution, thereby enhancing the catalytic performance.

## Introduction

Water electrolysis using renewable electricity has been regarded as a promising route to sustainable green hydrogen energy production^1–3^. Compared with traditional alkaline electrolysis, proton exchange membrane water electrolysis (PEMWE) has the advantages of faster dynamics, operation at higher current densities (maximum 2-3 A cm^−2^), and high-purity H_2_ production (>99.9999 vol%)^3–5^. However, the anodic oxygen evolution reaction (OER) is a four-proton-coupled-electron transfer process, which is the efficiency bottleneck of the water-splitting reaction due to a higher reaction energy barrier than the hydrogen evolution reaction (HER). In addition, the harsh acidic and strong oxidative operating conditions impose a huge challenge to the development of high-performance electrocatalysts for the acidic OER process, which hinders the widespread application of PEMWE^6,7^. More importantly, the use of abundant natural seawater as an electrolyte in water-splitting devices is highly desired but remains challenging due to the progressively increasing surface acidity at the anode due to the hydroxide removal during OER and hypochlorite formation^8,9^. Therefore, overcoming the activity/stability trade-off of acidic OER catalysts can also significantly address the challenges of natural seawater electrolysis.

For decades, Ir- and Ru-based materials have been considered as benchmark electrocatalysts to balance the stability and activity in acidic electrolyte^10,11^. However, the Ir-based materials suffer from low mass activity and high cost (US$60,670 kg^−1^), which has severely hindered its practical applications in a large scale; while Ru (US$9,523 kg^−1^) is 1/6 of the cost of Ir and typically exhibits higher intrinsic activity, making the Ru-based catalysts as a more ideal candidate to balance cost and activity in commercial acid-based devices^7,12–14^. Despite the above advantages, the use of Ru-based catalysts for acidic OER is hampered by the poor stability (<100 hours at 10 mA cm^−2^) in acidic conditions due to the over-oxidation of Ru to high valence Ru^n+^ (n > 4) materials (e.g. RuO_4_) in highly oxidative environments, which inevitably leads to the collapse of the crystal structure^4,5,10,11^, dissolution of active sites and thus deterioration of catalytic performance^4–7,10–14^.

Therefore, to achieve high-performance OER over Ru-based catalysts, a trade-off between the activity and the stability is highly desirable and yet challenging. Recently, much effort has been devoted to improving the acidic OER performance of Ru-based electrocatalysts via strategies such as alloying^15,16^, defect engineering^3,5,7,14,17^, strain effect^4,18^, structure tuning^13,14,19^ and so on. These strategies aim to prevent excessive oxidation and dissolution of Ru active sites by tuning the electronic structure of Ru, resulting in optimized OER activity and stability compared to commercial RuO_2_ nanoparticles in acids. Nevertheless, most of the reported catalysts are powder-based and need to be loaded onto substrates with the assistance of binders to prepare working electrodes^4–7,12–19^. The catalysts requiring binders suffer from high mass transport resistance, severe performance degradation, and easy delamination, especially at high current densities^20,21^. Therefore, most of the Ru-based catalysts reported to date can only operate at low current densities (<100 mA cm^−2^)^3–7,13–17,19^, and the stability remains limited to within tens of hours at low current densities ( ~ 10 mA cm^−2^)^4,5,12–14,17–19^, which is far from meeting the industrial application requirements.

In this contribution, we report a one-step method for the in situ growth of TiO_x_ nanorods on a Ti foam substrate while reducing Ru^3+^ to small-sized Ru nanoparticles anchored thereon (Ru/TiO_x_) as a robust and efficient binder-free electrode for OER in acidic media. By applying this cost-effective method, which is free of additional Ti source, reducing agent, and binder, we were able to construct intrinsic defects in the TiO_x_ (*x* denotes the non-stoichiometric compound resulting from oxygen vacancies), which stabilized the highly active Ru sites through the enhanced metal-support interaction. As a result, the Ru/TiO_x_ exhibits low overpotentials of 174, 209, and 265 mV to achieve current densities of 10, 100, and even a high current density of 500 mA cm^−2^ in a challenging acid electrolyte, respectively. The non-stoichiometric oxide ensures the charge accumulation at the Ru site, resulting in a significant improvement of OER activity (2.9 times) and stability (10.0 times), compared to the stoichiometric RuO_x_/TiO_2_. More importantly, Ru/TiO_x_ can be directly applied to the electrolysis of pure natural seawater, requiring an overpotential of 320 mV to achieve a high current density of 100 mA cm^−2^, suggesting its great potential for practical seawater-splitting applications. Therefore, we offer an integrated preparation strategy to overcome the activity/stability limitation of Ru-based catalysts. The non-stoichiometric compound-induced charge redistribution at metal sites demonstrated in this work may pave wide avenues for the development of promising catalysts for practical PEMWEs and other acid-based devices.

## Results

### Synthesis and characterization of catalysts

**Fig. 1:**
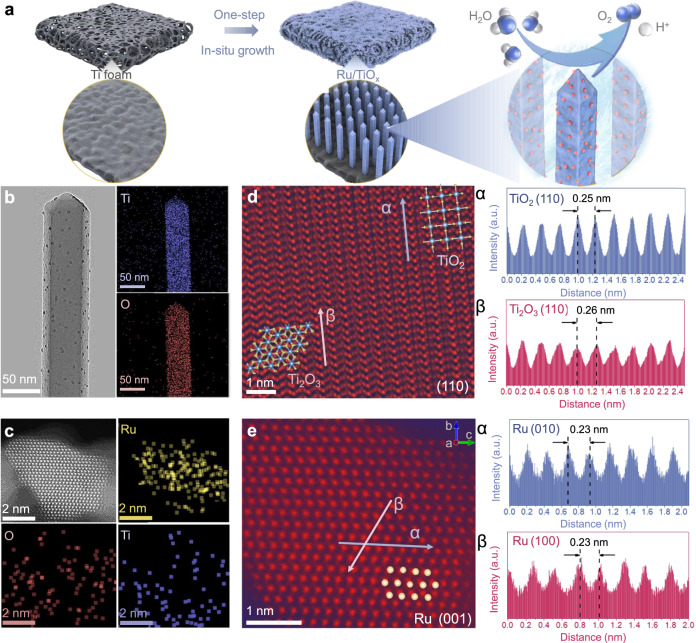
Morphology and structure characterizations of as-synthesized Ru/TiO_x_. **a** Schematic illustration of synthesis of Ru/TiO_x_ as binder-free electrode towards oxygen evolution reaction (OER) in acidic media. **b** Transmission electron microscopy (TEM) image of Ru/TiO_x_ and corresponding energy dispersive X-ray spectroscopy (EDS) mapping profile of Ti (blue) and O (red). **c** Aberration-corrected high angle annular dark field-scanning TEM (HAADF-STEM) image of Ru/TiO_x_ and corresponding EDS mapping profile of Ru (yellow), Ti (blue) and O (red). **d**, **e** Aberration-corrected HAADF-STEM images of TiO_x_ support (**d**) and Ru site of Ru/TiO_x_ (**e**). STEM intensity profiles are presented to directions labeled with blue (α) and red (β) arrows. (α and β) show the corresponding intensity profiles and the lattice fringe spacing.

The synthesis process of the self-supported Ru/TiO_x_ electrode is schematically shown in Fig. 1a. Through a facile hydrothermal process, the in situ growth of TiO_x_ and the loading of Ru nanoparticles (NPs) are realized in one step. Specifically, commercial Ti foam (TF) with a porous structure and good chemical/mechanical stability is selected as both substrate and Ti source for TiO_x_ growth. The Ti foam serves as both substrate and reducing agent to efficiently reduce Ru^3+^ ions through a redox interaction-engaged strategy due to a thermodynamically favorable process. Photographs (Supplementary Fig. 1) show that Ru/TiO_x_ is completely covered on TF, and the color of TF changes from gray to dark. Typically, aqueous solutions of RuCl_3_ and HCl were heated up to 200 °C for 20 h. HCl not only acted as a chemical corrosive agent to induce defects on the TF surface, which enabled the growth of rutile phase nanorods, but also provided an acidic condition to etch the TF and form titanium ions (Ti^3+^), which were transformed into titanium (III) (hydro)oxide nanostructure via Ti^3+^ hydrolysis in acidic aqueous solution during the hydrothermal treatment^22^. Subsequently, when the metal ions (Ru^3+^) came into contact with the titanium(III) (hydro)oxide nanostructure, which had a strong reducing ability, they were immediately reduced by the Ti^3+^ and rapidly nucleated on the as-formed TiO_2_ nanorod surfaces to grow into clusters and further into nanoparticles. Meanwhile, the support was partially oxidized by the metal ions and further converted into TiO_x_, resulting in Ru/TiO_x_ nanocomposites^23,24^.

Field emission scanning electron microscopy (FESEM; Supplementary Figs. 2, 3) reveals the 3D porous structure of pristine TF and the successful growth of TiO_x_ nanorod arrays. Transmission electron microscopy (TEM) and corresponding energy dispersive X-ray spectroscopy (EDS) mapping profiles reveal the high dispersion of Ru NPs, and homogeneous distribution of Ti and O elements throughout the Ru/TiO_x_ sample (Fig. 1b). According to high-resolution TEM (HRTEM; Supplementary Fig. 4), Ru NPs with ~ 4 nm are homogeneously deposited on TiO_x_, which has a rod-like structure with pyramidal morphology consisting of dominantly exposed reductive lateral facets (110) and oxidative top facets (111). The anisotropic growth of rutile nanocrystals along the [001] direction was caused by excess Cl^−^ preferentially adsorbed on the (110) plane of the rutile TiO_2_ surface, which suppressed grain growth along the [110] direction and accelerated it in the [001] direction^22,25^. The *d*-spacing of the nanorods is determined to be 0.32 nm, corresponding to the (110) crystal plane fringes of the rutile TiO_2_; the *d*-spacing of the nanoparticles is determined to be 0.21 nm, attributed to the Ru (101) plane, which agrees well with the negligible broad peaks of Ru due to the high Ru dispersion in the X-ray diffraction (XRD) pattern (Supplementary Fig. 5). The high-angle annular dark-field scanning TEM (HAADF-STEM) image and the intensity maps clearly show the atomic arrangement of Ru, in which the lattice space of 0.23 nm corresponds to the Ru {100} family of crystal planes (Fig. 1e and Supplementary Fig. 6). Combined with STEM-EDS (Fig. 1b, c), it can be explained that Ru NPs with the size of ~4 nm are homogeneously deposited on in situ formed rutile TiO_x_ nanorod by one-step hydrothermal process. Interestingly, it is found that the atomic arrangement at the edge of TiO_x_ nanorod is different from that of the body part. As shown in Fig. 1d, rutile-type TiO_2_ (above the interface) is in the body part of the nanorod, while Ti_2_O_3_ (lies below) is at the edge, assuming a zigzag configuration, which is typical of corundum-type structure^26–28^. According to the previously proposed growth mechanism, the redox reaction between titanium(III) (hydro)oxide and Ru ions during the hydrothermal process may be responsible for the formation of the Ti_2_O_3_ phase at the edge^24^. For comparison, we synthesized TiO_2_ on TF substrate using the same method (except for the addition of Ru source). The FESEM images show the nanorod structure of TiO_2_/TF with flat apex, which is different from that of the Ru/TiO_x_ with pyramidal apex (Supplementary Fig. 7). The HAADF-STEM image and the corresponding fast Fourier transforms (FFTs) show the rutile-type TiO_2_ with (101) crystal plane in both the body and the edge part of the nanorod (Supplementary Fig. 8). Therefore, the redox reaction during the hydrothermal process results in the formation of a highly active edge part of the nanorod, which leads to the coexistence of TiO_2_ and Ti_2_O_3_ in the as-synthesized sample, which we denoted as Ru/TiO_x_. It is reported that the substoichiometric phase Ti_2_O_3_ exhibits high electrical conductivity and the rutile-corundum interface is favorable for charge transfer, which is beneficial in energy storage systems and electronic devices^26–28^. Therefore, we believe that the in situ formed Ru/TiO_x_ with supported acid-stable Ti framework, ultrafine nanoparticles, and rutile-corundum interface can be a good candidate for OER electrocatalysis in acidic media.

### Electrocatalytic OER performance in acidic media

**Fig. 2:**
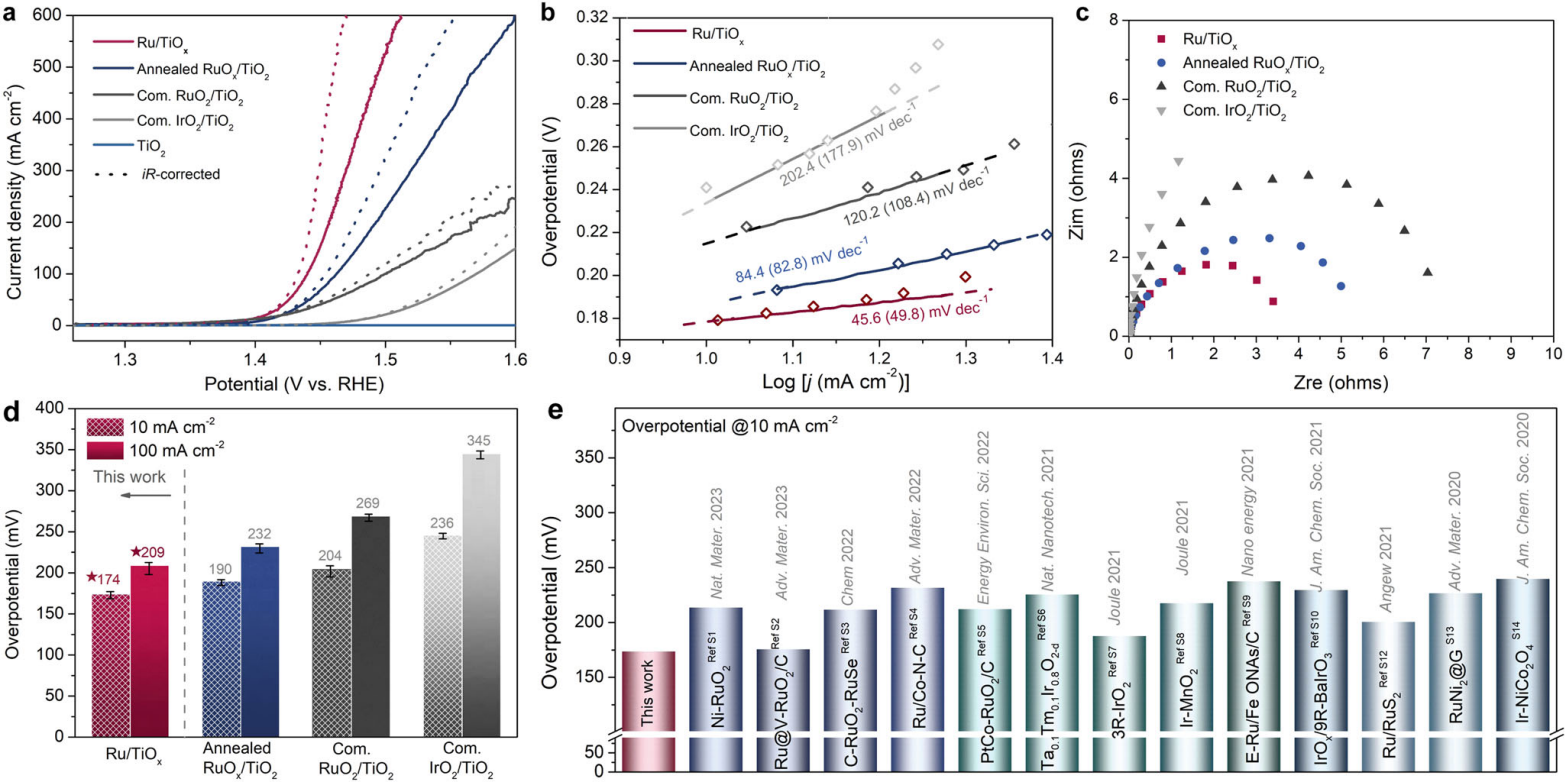
Electrocatalytic oxygen evolution reaction (OER) activity in 0.5 M H_2_SO_4_ solutions (pH = 0.3). **a** Linear sweep voltammetry (LSV) curves (both *iR*-corrected and *iR*-free) of Ru/TiO_x_, annealed RuO_x_/TiO_2_, com. RuO_2_/TiO_2_, com. IrO_2_/TiO_2_ and TiO_2_. (Com. RuO_2_/TiO_2_ and com. IrO_2_/TiO_2_ denotes commercial RuO_2_/TiO_2_ and commercial IrO_2_/TiO_2_, respectively). *iR* is automatically compensated by workstation (95%). Mass loadings of noble metals are 0.0715, 0.0867, 0.0992 and 0.1135 mg cm^−2^ for Ru/TiO_x_, annealed RuO_x_/TiO_2_, com. RuO_2_/TiO_2_ and com. IrO_2_/TiO_2_, respectively. **b** Tafel plots derived from the LSV curves (solid line) and the steady-state polarization curves (scatters) (values in parentheses were derived from steady-state polarization curves). **c** Electrochemical impedance spectroscopy (EIS) of Ru/TiO_x_, annealed RuO_x_/TiO_2_, com. RuO_2_/TiO_2_ and com. IrO_2_/TiO_2_. **d** Comparison of overpotentials without *iR* correction at 10 and 100 mA cm^−2^ for Ru/TiO_x_, annealed RuO_x_/TiO_2_, com. RuO_2_/TiO_2_ and com. IrO_2_/TiO_2_. (Error bar: standard error of three repeated measurements). **e** Comparison of the overpotentials of Ru/TiO_x_ and state-of-the-art Ru/Ir-based electrocatalysts at 10 mA cm^−2^ in acidic media.

The catalytic performances of self-supported Ru/TiO_x_ towards OER were directly evaluated in 0.5 M H_2_SO_4_. For comparison, annealed RuO_x_/TiO_2_, TiO_2_/TF, commercial RuO_2_/TiO_2,_ and IrO_2_/TiO_2_ electrocatalysts were also investigated under the same conditions. We compare the OER activity in three prevalent metrics, i.e., geometric activity (*j*_*geo*_, geometric area of the working electrode), specific activity (*j*_*s*_, electrochemical active surface area (ECSA)), and mass activity (*j*_*m*_, mass of metals). Geometric activity is important at the device level. As can be seen from the *iR*-free linear sweep voltammetry (LSV) curves, Fig. 2a, the Ru/TiO_x_ exhibits the highest performance of all samples, requiring overpotentials of only 174, 209, and 226 mV to achieve current densities of 10, 100, and even 200 mA cm^−2^_geo_, respectively, significantly outperforming the other control samples, including the state-of-the-art commercial RuO_2_/TiO_2_ and IrO_2_/TiO_2_. To eliminate electrolyte resistance, the LSV curves with 95 % *iR*-compensation were also shown in Fig. 2a. The measured overpotentials before and after *iR* correction are summarized in Supplementary Fig. 9. Supplementary Movie 1 shows the process of the binder-free Ru/TiO_x_ electrode working directly as an anode for water electrolysis at different current densities. It can be observed that vigorous oxygen bubbles are rapidly released from the anode. In addition, under the harsh acidic and oxidative environment, no catalyst is detached from the electrode during the whole process of the stability test, proving its superiority in high current density electrolysis. The trend of overpotentials to achieve a high current density of 100 mA cm^−2^ is ranked in the following order: Ru/TiO_x_ (209 mV) <annealed RuO_x_/TiO_2_ (232 mV) <commercial RuO_2_/TiO_2_ (269 mV) <commercial IrO_2_/TiO_2_ (345 mV) (Fig. 2d). The bare in situ formed TiO_2_ nanorods grown on TF (TiO_2_/TF) under the same hydrothermal conditions showed negligible OER performance, while commercial RuO_2_/TiO_2_ (*η*_10_ = 204 mV) showed improved electrocatalytic activity than commercial RuO_2_/TF (*η*_10_ = 242 mV) at the same loading, suggesting that TiO_2_ itself is inert in OER but plays an important role in supporting and dispersing active sites (Supplementary Fig. 10). Compared with the TiO_2_-based samples, the physically-adsorbed RuO_2_/TiO_2_ shows significantly lower OER activity than both the in situ formed Ru/TiO_x_ and the annealed RuO_x_/TiO_2_ electrode, demonstrating the superiority of the in situ growth strategy, which results in a strong internal interaction between Ru and TiO_x_, thereby optimizing the intrinsic activity of each active site. The OER performance of the Ru/TiO_x_ is firstly compared in terms of overpotential to reach 10 mA cm^−2^. The OER activity of Ru/TiO_x_ is substantially superior to that of most Ru/Ir-based electrocatalysts reported in the literature (Fig. 2e and Supplementary Table 1), including recently reported representative electrocatalysts Ni-RuO_2_ (214 mV)^3^, Ru/Co-N-C (232 mV)^29^, etched-Ru/Fe oxide nano assemblies (238 mV)^30^, Ru@IrO_x_ (282 mV)^31^, RuNi_2_@graphene (227 mV)^12^, and so on. More importantly, the excellence of electrocatalytic performance of Ru/TiO_x_ is more pronounced at high current densities. For example, the Ru/TiO_x_ electrode requires an overpotential of only 265 mV to achieve a high current density of 500 mA cm^−2^, which is required by the industrial criteria but rarely achieved in the acidic OER process^3–7,13–17,19^. It is well known that the specific activity is mainly determined by the ECSA of the catalysts. In order to evaluate the corresponding intrinsic activity, the electrochemical double-layer capacitance (*C*_*dl*_) was used to compare the order of the ECSA. Clearly, Ru/TiO_x_ exhibits a much higher *C*_*dl*_ value of 13.73 mF/cm2, revealing the exposure of more active sites for OER (Supplementary Fig. 11). We further normalized the LSV curves with ECSA to compare the specific activity of the electrocatalysts (Supplementary Fig. 12a). Interestingly, the specific activities and the geometric activity show the same trend (Supplementary Fig. 12b). The Ru/TiO_x_ can reach a high *j*_*ECSA*_ of 1.49 mA cm^−2^ at 1.50 V vs. RHE, which is about 2.5 times that of the Com-RuO_2_/TiO_2_ (about 0.60 mA cm^−2^), indicating its superior intrinsic activity for the OER. To quantitatively evaluate the mass activity, we determined the corresponding mass loading of the Ru/TiO_x_ and other control samples by inductively coupled plasma mass spectrometry (ICP-MS) measurement (Supplementary Table 2). As shown in Supplementary Fig. 13, the mass activity of Ru/TiO_x_ (2128.2 A g _Ru_^−1^) at the potential of 1.45 V vs. RHE is 4.6 and 46.2 times that of Com-RuO_2_/TiO_2_ (462.0 A g _Ru_^−1^) and Com-IrO_2_/TiO_2_ (46.0 A g _Ir_^−1^), respectively. These results further substantiated the remarkable electrocatalytic performance of Ru/TiO_x_ compared to the other reported catalysts (Supplementary Table 3). The intrinsic activities of Ru/TiO_x_ were further assessed based on turnover frequencies (TOFs) at different overpotentials, which are among the highest when compared with representative OER catalysts in various acidic media (Supplementary Fig. 14a and Table 4). For example, the TOF of Ru/TiO_x_ is calculated to be 1.960 s^−1^ at an overpotential of 300 mV based on the total loading mass, which increases to 2.192 s^−1^ when calculated based on ECSA (Supplementary Table 5). We also provided a bar graph of ECSA-normalized current densities and TOF values at an overpotential of 300 mV, which shows a similar activity trend (Supplementary Fig. 14b). The above results prove that Ru/TiO_x_ shows the highest intrinsic activity per site. In order to evaluate the catalytic kinetics of OER, Tafel plots are obtained based on the *iR*-free LSV curves and the steady-state polarization curves (Fig. 2b and Supplementary Fig. 15)^14^, where Ru/TiO_x_ exhibits the lowest Tafel slope of 45.6 (49.8) mV dec^−1^, indicating the highest charge transfer efficiency and fastest reaction rate among these prepared samples. The electrochemical impedance spectroscopy (EIS) measurements also confirm a faster OER process of Ru/TiO_x_, as evidenced by its remarkably lower charge transfer resistance (*R*_*ct*_) than that of other control samples (Fig. 2c). From the above electrochemical results, it is clear that the well-aligned Ru/TiO_x_ nanoarrays play an important role in the catalyst-liquid/gas interaction at the interfaces and consequently in the OER performance, especially at high current densities. To quantitatively analyze the differences between the TF and the Ru/TiO_x_, we measured the liquid contact angles (LCA) and bubble contact angles (BCA) on their surfaces, respectively. The well water adsorption originating from the Ru/TiO_x_ nanoarrays significantly improves the surface wettability and then favors the contact between the electrolytes and the electrode surface, as evidenced by the decreased LCA from 43.6° (TF) to 0° (Ru/TiO_x_) (Supplementary Fig. 16). In addition, the vertically aligned nanoarrays result in effective gas escape, especially at high current densities, due to the discontinuous state of the three-phase gas-solid-liquid contact line between the bubbles and the electrode surfaces, which contributes to the exceptionally small contact area and lower adhesion force^32^. Therefore, the Ru/TiO_x_ shows a higher BCA of 151.3°compared to the TF (Supplementary Fig. 17), which shows a more tremendous potential to release the as-formed O_2_ bubbles with superfast speed and prevent bubble retention.

**Fig. 3:**
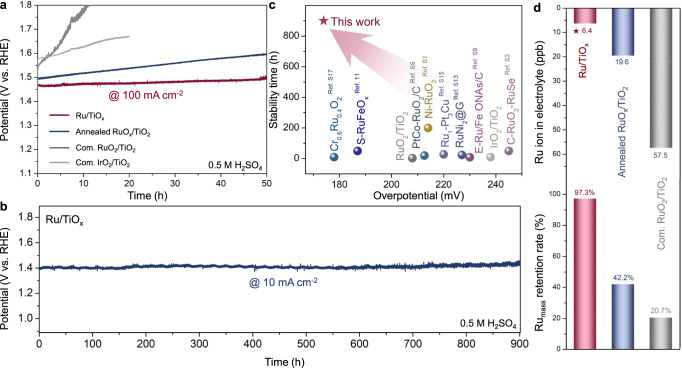
Electrocatalytic OER stability in 0.5 M H_2_SO_4_ solutions (pH = 0.3). **a** Chronoamperometric curves of Ru/TiO_x_, annealed RuO_x_/TiO_2_, com. RuO_2_/TiO_2_ and com. IrO_2_/TiO_2_ for OER at 100 mA cm^−2^. The chronoamperometric curves are not *iR* compensated. Mass loadings of noble metals are 0.0715, 0.0867, 0.0992, and 0.1135 mg cm^−2^ for Ru/TiO_x_, annealed RuO_x_/TiO_2_, com. RuO_2_/TiO_2_ and com. IrO_2_/TiO_2_, respectively. **b** Chronoamperometric curves of Ru/TiO_x_ for OER at 10 mA cm^-2^. **c** Comparison of the overpotential and stability time of Ru/TiO_x_ with state-of-the-art OER electrocatalysts in acidic media. **d** Inductively coupled plasma-mass spectrometry (ICP-MS) analysis for dissolved Ru ions in post chronopotentiometry electrolyte and Ru mass percentage retained in Ru/TiO_x_, annealed RuO_x_/TiO_2_ and com. RuO_2_/TiO_2_ catalyst after the chronoamperometric test.

Long-term stability under high current density is another essential standard for evaluating industrial applications, which is particularly critical for acidic OER electrocatalysts due to the highly corrosive electrolytes and oxidative operating conditions. Thus, the OER stability of the as-prepared Ru/TiO_x_ was evaluated by continuous cyclic voltammograms (CVs) up to 550 mA cm^−2^ for 50 cycles and chronopotentiometry test at constant current densities of 10 mA cm^−2^ and 100 mA cm^−2^. As shown in Supplementary Fig. 18, no obvious decay in polarization curves was observed for Ru/TiO_x_ after 50 OER cycles up to 550 mA cm^−2^, suggesting its durability under large current densities. Furthermore, the overpotential required to achieve the current density of 100 mA cm^−2^ remained constant with a negligible increase of 28 mV over 50 h (Fig. 3a). In contrast, the annealed RuO_x_/TiO_2_ electrode exhibited a continuously increasing overpotential of 92 mV until the final activity degradation, while the commercial RuO_2_/TiO_2_ and IrO_2_/TiO_2_ underwent severe OER performance decay within only 20 h. Similar to the control samples, many other reported catalysts suffer from poor stability of only a few hours at low current density (10 mA cm^−2^) due to the loss of active phase under this harsh condition (Supplementary Fig. 19 and Table 1). However, the as-synthesized Ru/TiO_x_ showed remarkable long-term stability for OER at the current density of 10 mA cm^−2^. A nearly horizontal line with only a 20 mV increase in overpotential was obtained after 900 h of continuous CP test, maintaining 98.6% of the initial activity, further confirming the high stability of our catalysts in acidic environment (Fig. 3b). Notably, the low overpotential of 174 mV and the long-term durability of 900 h at 10 mA cm^−2^ for the Ru/TiO_x_ surpasses most of the reported acidic OER electrocatalysts (Fig. 3c and Supplementary Table 1).

ICP-MS analysis and X-ray photoelectron spectroscopy (XPS) were further performed to determine the amounts of dissolved Ru ions in the electrolytes after the stability test and the Ru content remained in the catalysts (Fig. 3d). For the as-synthesized Ru/TiO_x_, the extremely low Ru ion concentration (6.4 ppb) in the electrolyte after the stability test and the maintenance of the Ru content (97.3%) in the catalyst manifest the effective protection of Ru sites from dissolution (Fig. 3d and Supplementary Table 2). For comparison, only 42.2% and 20.7% Ru remained in the annealed RuO_x_/TiO_2_ and commercial RuO_2_/TiO_2_ catalyst, proving the stability of the Ru sites in the Ru/TiO_x_ catalyst, consistent with the trend obtained by XPS results (Supplementary Fig. 20 and Table 2).

To illustrate the practical capabilities of Ru/TiO_x_ in water splitting, a PEMWE was constructed to evaluate the performance under conditions that are representative of industrial applications (Supplementary Figs. 21 and 22). As shown in Supplementary Fig. 22a, the PEMWE with Ru/TiO_x_ as an anode shows 1.71 V at 1 A cm^−2^, which is 0.23 V lower compared with RuO_2_ anode. Besides, the PEMWE with Ru/TiO_x_ as an anode well maintains its voltage (Δ*E* < 0.01 V) during 200 h operation at 500 mA cm^−2^, comparable to the recently reported catalysts (Supplementary Fig. 22b and Table 6). In addition, the stability significantly outperforms those with RuO_2_ anode, which shows Δ*E* > 0.2 V decay within 100 h (Supplementary Fig. 22b), further highlighting the superiority of self-supported Ru/TiO_x_ in OER at high current densities.

### Electrocatalytic OER performance in natural seawater

In particular, in the seawater electrochemical splitting process, the pH at the anode decreases dramatically, resulting in a local acidic environment and deactivation of most electocatalysts^8,33^. Therefore, an additional buffer solution such as KOH is required to mitigate the corrosion and dissolution of the catalyst caused by this local acidity in the seawater electrolysis^34,35^. However, the use of such chemical agents increases the excess consumption and cost, especially in large-scale applications. From a green and sustainable point of view, it is highly desirable to directly use abundant natural seawater without any treatment in water electrolyzer as an energy-efficient technology, but it still remains a great challenge. Therefore, the search for an active and durable anode material that can survive in natural seawater, rather than buffered or simulated seawater, is of great importance^33^.

As a binder-free electrode, the as-synthesized Ru/TiO_x_ exhibits good activity and durability under acidic and oxidizing conditions as concluded above. Based on the reduced-pH at the anode during the electrolysis of seawater, we believe that such a stable structure can also prevent the loss of catalytic sites in a similar local-acidic environment during OER in real seawater without any buffer solutions. As expected, the Ru/TiO_x_ exhibited similarly high OER performance in real seawater (Yellow Sea, China) without any further treatment, requiring an overpotential of 320 mV to achieve a high current density of 100 mA cm^−2^ (Supplementary Fig. 23a). More importantly, the Ru/TiO_x_ maintained the initial high catalytic activity in seawater even after prolonged use. As shown in Supplementary Fig. 23b and Movie 2, Ru/TiO_x_ can deliver a 100 mA cm^−2^ at a constant potential of 1.57 V vs. RHE. Although there are minor fluctuations during the reaction process due to the complex composition of the unpurified and unbuffered seawater electrolyte (Supplementary Table 7), the performance remained almost unchanged at the end of the 20 h reaction. It is noteworthy that almost all HER and OER catalysts reported in seawater electrolysis require an additional buffer solution^34,35^. Even the very few reported catalysts that can be used in buffer-free seawater cannot achieve a high current density and remain stable for such a long time^9,36^. Therefore, the as-prepared catalyst represents a breakthrough in the direct utilization of natural water resources, which has great potential for large-scale applications of seawater-based energy storage and conversion devices.

### Origin of improved electrocatalytic OER performance

To elucidate the origin of the enhanced OER catalytic performance of the Ru/TiO_x_ in an acidic environment, we synthesized two control samples of annealed RuO_x_/TiO_2_ and physically mixed RuO_2_/TiO_2_ with the same loading. The above results indicate that the two control samples exhibited apparently low electrocatalytic activity and stability in an acidic solution compared with those of Ru/TiO_x_. This motivates us to investigate the differences in the microstructure and electronic structure of Ru/TiO_x_, RuO_x_/TiO_2_, and RuO_2_/TiO_2_, which are closely related to the electrocatalytic performance for acidic OER. The chemical states for Ru, Ti, and O in the three samples before and after the OER stability test were first investigated by XPS analysis.

**Fig. 4:**
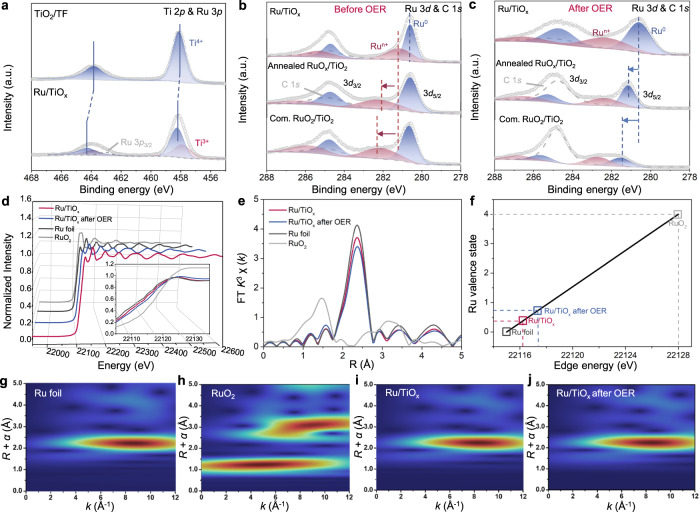
Electronic structure analysis of Ru/TiO_x_. **a** XPS of Ti 2*p* and Ru 3*p* for the bare TiO_2_ and Ru/TiO_x_. **b,** **c** Ru 3*d* XPS spectra of Ru/TiO_x_, annealed RuO_x_/TiO_2_ and com. RuO_2_/TiO_2_ before OER stability test (**b**) and after OER stability test (**c**). **d** Ru *K*-edge synchrotron-based X-ray absorption near-edge structure (XANES) spectra of Ru/TiO_x_ before and after OER stability test using Ru foil and commercial RuO_2_ as references. **e** Fourier-transformed (FT) *k*3-weighted *χ(k)*-function of the extended X-ray absorption fine structure (EXAFS) spectra for the Ru *K*-edge. **f** Relation between the Ru *K*-edge absorption energy (*E*_*0*_) and valence states for Ru/TiO_x_, Ru/TiO_x_ after OER stability test, Ru foil, and RuO_2_. **g**–**j** Wavelet transforms for the *k*3-weighted EXAFS signals of Ru foil (**g**), RuO_2_ (**h**), Ru/TiO_x_ (**i**) and Ru/TiO_x_ after OER stability test (**j**).

In particular, the in situ formation of Ru/TiO_x_ via one-step hydrothermal process induces a strong interaction between the Ru sites and the TiO_x_ support, as evidenced by the positive core-level shift in the binding energies of Ti 2*p* and O 1 *s* compared to bare TiO_2_/TF (Fig. 4a and Supplementary Fig. 24). This interaction allows the generation of Ti^3+^ defects and oxygen vacancies, which are not only beneficial for preventing the oxidation of metallic Ru to soluble oxidized species (Ru^>4+^), but also favorable for bonding with water molecules, promoting the water splitting process^9,37,38^. It should be noted that the annealing of Ru/TiO_x_ resulted in the formation of RuO_x_ with a mostly oxidized surface and the complete transformation of Ti_2_O_3_ to TiO_2_, as indicated by the positive core-level shift of the binding energies of Ru 3*d* and Ti 2*p* (Fig. 4b and Supplementary Fig. 25). These changes suggest that the removal of the Ti^3+^ defects and oxygen vacancies weakened the interaction between Ru and TiO_x_, which led to the low electrocatalytic activity and stability of the annealed RuO_x_/TiO_2_^37^.

To further verify the high stability of Ru/TiO_x_ for OER in the acidic electrolyte, the chemical states for Ru, Ti, and O in the three samples after the acidic OER stability tests were analyzed and compared with those before OER (Fig. 4c, Supplementary Table 8). For Ru/TiO_x_, the peak at 280.61 eV for Ru 3*d*_5/2_ (Ru^0^) remained generally unchanged as compared with that of the sample before the OER test (280.60 eV), while the peak at 281.2 eV for Ru 3*d*_3/2_ (Ru^n+^, n < 4) slightly shifted to higher binding energies, indicating that the active Ru sites were only partially oxidized but mainly remained in the low-valence state (Ru^n+^, n < 4) during the 50 h OER test (Fig. 4c). For comparison, the changes for annealed RuO_x_/TiO_2_ and commercial RuO_2_/TiO_2_ are much more significant: the peaks for Ru^0^ and Ru^4+^ shift positively for 0.54 and 0.90 eV, respectively, indicating that the active species Ru were all over-oxidized to Ru^n+^ (n > 4), which were easily separated from the catalysts and dissolved during the reaction process, leading to the degradation of the OER performance. The Ru^n+^/Ru^0^ ratios calculated from the corresponding peak area in the Ru 3*d* XPS spectra indicate that the oxidation state of Ru in the as-prepared catalysts follows the trend of commercial RuO_2_/TiO_2_ > annealed RuO_x_/TiO_2_ > Ru/TiO_x_, which is opposite to the trend of OER stability (Supplementary Fig. 26). The established valence-stability relationship proved that the low-valence Ru in Ru/TiO_x_, due to the strong interaction between Ru and TiO_x_, is highly active and stable, further highlighting the advantages of the in situ and one-step growth strategy. Afterwards, the chemical environments of Ti and Ru in the Ru/TiO_x_ catalyst under actual OER conditions were monitored by in-situ XPS (Supplementary Figs. 27–29 and Table 9). Significantly, the Ru 3*d* XPS peaks at 280.1 and 284.2 eV exhibit negligible changes with the applied potential increased from 1.0 to 1.7 V vs. RHE (Supplementary Fig. 28a and Table 9). Detailed quantitative analysis shows the ratio of Ru^n+^/Ru^0^ remains almost identical at 0.7 as the voltage increases, which is consistent with the ex-situ XPS analysis results (Supplementary Figs. 26 and 29). The above results further confirm the stable Ru chemical state in Ru/TiO_x_ during the OER process. For the Ti 2*p* spectra (Supplementary Figs. 28b and Table 9), the coexistence of Ti^3+^ and Ti^4+^ species was distinguished. Interestingly, as the voltage increases, the ratio of Ti^3+^/Ti^4+^ slightly decreases and remains stable at 0.17, indicating the critical role of the Ti^3+^ in stabilizing Ru active sites. Corresponding to this phenomenon is the O_V_/O_L_ (oxygen vacancy/lattice oxygen) ratio obtained from the O 1 *s* spectra (Supplementary Figs. 28c and 29). It can be seen that as the voltage increases, the O_V_/O_L_ ratio first decreases and then almost returns to the initial state, manifesting that O_V_ regeneration is accompanied by the release of oxygen, thereby stabilizing active species^39^.

X-ray absorption spectroscopy (XAS) is also used to precisely investigate the electronic structure of Ru in the Ru/TiO_x_. In detail, the X-ray absorption near edge structure (XANES) spectra (Fig. 4d) show that the adsorption threshold position is close to that of Ru foil but remarkably different from that of RuO_2_, indicating that metallic Ru NPs are dominant in Ru/TiO_x_. In addition, the Ru *K*-edge spectra before and after the OER reaction are quite similar (Fig. 4b–d), indicating that the oxidation state of Ru is stable, as suggested by the XPS results. Specifically, the Ru valence states are quantitatively measured by the adsorption energy (*E*_*0*_) of the Ru/TiO_x_ before and after OER (Fig. 4f and Supplementary Table 10). After the OER test, the *E*_*0*_ of Ru/TiO_x_ was positively shifted to higher energy compared to that of pristine Ru/TiO_x_, with the oxidation state of Ru slightly increased from +0.34 to +0.72 during electrocatalysis, which is still far below the +4 valence state. Therefore, the strong interaction between Ru and TiO_x_ effectively suppresses the over-oxidation of Ru during the OER, resulting in high catalytic stability. Subsequently, the corresponding Fourier transforms of the extended X-ray absorption fine structure (EXAFS) spectra show that the length of Ru-Ru bonds in Ru/TiO_x_ is similar to that of Ru foil, which shows the dominant Ru-Ru scattering path (Fig. 4e)^38^. The coordination numbers and interatomic distances were determined from the EXAFS fitting results (Supplementary Fig. 30 and Table 11). Compared to the pristine Ru/TiO_x_, the Ru-Ru interatomic distance slightly increased, while the Ru-O interatomic distance slightly decreased, presumably due to the partial structural disorder of Ru/TiO_x_ after OER^17^. The wavelet transforms (WT) of the Ru *K*-edge EXAFS oscillations show that the coordination features of Ru in Ru/TiO_x_ are similar to those in Ru foil, with a predominant Ru-Ru coordination at 2.3 Å, indicating strong metallic coordination in the Ru/TiO_x_ (Fig. 4g–j). Thus, the enriched electrons are transferred from TiO_x_ to the empty *d* bands of the confined Ru NPs, which stabilizes the coordination environment of Ru and prevents it from further oxidation.

The nanostructure integrity of an electrocatalyst at the atomic scale is also essential for achieving high acid OER performance. As shown in Supplementary Fig. 31, the nanorod-structure of Ru/TiO_x_ was well maintained after both the 900 h and 50 h stability tests, which is attributed to the mechanical and chemical stability of the TiO_x_ support. Furthermore, the nanoparticle size of Ru slightly increased to ~6 nm with uniform distribution due to the strong interaction between Ru and TiO_x_ (Supplementary Fig. 32). In sharp contrast, for the annealed RuO_x_/TiO_2_, the TiO_2_ nanorods were intertwined and connected with each other with the boundary blurred after long-time OER reaction, while the edge collapsed, which failed to stabilize and confine the size of Ru nanoparticles, resulting in the overgrowth and easy dissolution of RuO_x_ active centers (Supplementary Figs. 33 and 34). In the commercial sample, the morphology collapsed and deactivated shortly after the OER process due to the weak binding of RuO_2_ and TiO_2_ by physical adsorption (Supplementary Fig. 33). Therefore, both the stable chemical states and well-maintained nanostructure contribute to the superior catalytic activity and long-term stability of Ru/TiO_x_ electrocatalysts in acidic electrolyte.

### Mechanism analysis of OER activity

**Fig. 5:**
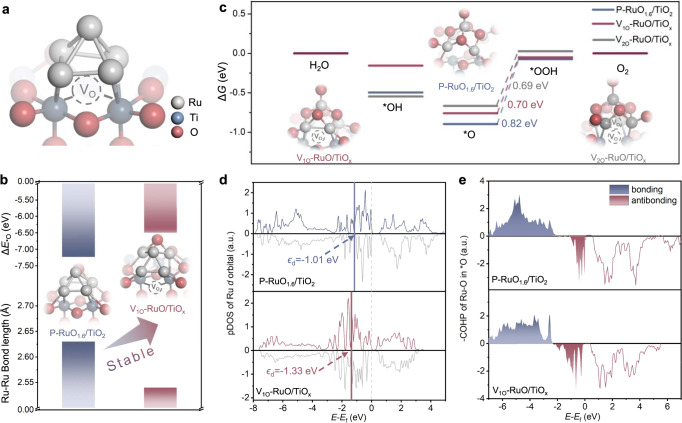
Mechanism analysis of Ru/TiO_x_ towards acidic OER. **a** Atomic structure of Ru_5_ cluster adsorbed on the TiO_2_ (110) surface with single oxygen vacancy (V_1O_-Ru_5_/TiO_x_). **b** The adsorption energy of oxygen (ΔE_*O_) on the Ru sites and the corresponding Ru-Ru bond length value (*d*_Ru-Ru_). The insets are the atomic structures of *O adsorbed on the structures of Ru_5_ cluster adsorbed on the pristine TiO_2_ (P-Ru_5_/TiO_2_) and V_1O_-Ru_5_/TiO_x_. **c** Gibbs free energy profile of OER for P-RuO_1.6_/TiO_2_, V_1O_-RuO/TiO_x_ and V_2O_-RuO/TiO_x_ (V_2O_ denotes 2 V_O_s). The insets are the atomic structures of V_1O_-RuO/TiO_x_ and V_2O_-RuO/TiO_x_. **d** Projected density of states (PDOS) and band center of Ru *d*-state for P-RuO_1.6_/TiO_2_ and V_1O_-RuO/TiO_x_. **e** Crystal Orbital Hamilton Population (COHP) for the adsorbed *O of Ru-O in P-RuO_1.6_/TiO_2_ and V_1O_-RuO/TiO_x_.

To further explore the possible catalytic mechanism on Ru/TiO_x_, the pH-dependence measurements of the corresponding OER activities were performed. As a result, the Ru/TiO_x_ shows pH-independent OER kinetics on the RHE scale, typical for AEM pathway (Supplementary Fig. 35). Density functional theory (DFT) calculations were performed to investigate the underlying mechanism of the superior OER performance of the Ru/TiO_x_ catalyst. The Pourbaix diagrams were constructed to study the oxidation states of Ru and Ti under different pH and potential conditions (Supplementary Fig. 36). From the calculated Pourbaix diagrams, it can be seen that the stable oxidation states of Ti and Ru are TiO_2_ and RuO_2_ under the experimental potential condition, respectively. To obtain stable Ru/TiO_x_ structures, we carried out ab initio molecular dynamics (AIMD) (Supplementary Figs. 37–40). The AIMD simulations were performed at 300 K for 10 ps with a time step 1 fs. These results provide the basis for our theoretical models. Our calculations focused on the Ru_5_ cluster adsorbed on the TiO_2_ (110) with one oxygen vacancy (V_O_) sites as the selected model structure (Fig. 5a), which is marked as V_1O_-Ru_5_/TiO_x_. By analyzing the results of Ru_5_ adsorbed on substrates with different V_O_ sites (Supplementary Fig. 39), we confirmed that the introduction of bridge V_O_ contributes to the stabilization of the Ru_5_ cluster by enhancing its adsorption strength. Specifically, when the Ru_5_ cluster is adsorbed on the nearest neighbor site of the bridge V_O_, the adsorption energy (E_ads_) decreases significantly from −2.54 eV to −3.94 eV compared to Ru_5_ adsorbed on pristine TiO_2_ (P-Ru_5_/TiO_2_). Moreover, the average bond length of the Ru-Ru bonds is reduced to 2.50 Å after the introduction of the V_O_ (Fig. 5b), which is consistent with the results of EXAFS analysis (Fig. 4e). The adsorption energy of oxygen (Δ*E*_O_) on the Ru sites can be a critical factor in determining the stability of the catalyst under acidic conditions^40^. As shown in Fig. 5b, the adsorption energy Δ*E*_O_ on the V_1O_-Ru_5_/TiO_x_ is 0.73 eV higher than that of the P-Ru_5_/TiO_2_, indicating that the introduction of V_O_ can improve the antioxidation ability of the Ru_5_ cluster in the V-Ru_5_/TiO_x_. It has been reported that catalysts tend to pre-adsorb oxygen species and generate amorphous nonstoichiometric oxide layers under the OER working condition^13,41^. Therefore, the Ru_5_ cluster will also be present as oxides during OER. Since the existence of oxygen vacancies will affect the oxidation state of Ru, we constructed different models (V_1O_-RuO/TiO_x_, V_2O_-RuO/TiO_x_, P-RuO/TiO_2_ and P-RuO_x_/TiO_2_, V_2O_ denotes two V_O_) to study the OER process (Supplementary Fig. 40). Whether it is the initial Ru_5_ cluster or RuO_x_ after pre-oxidation, the introduction of V_O_ helps to maintain the Ru atom at the interface in a lower valence state. Bader charge analysis shows that the charge of the Ru atom at the interface increases from 7.72 e^−^ in P-Ru_5_/TiO_2_ to 7.87 e^−^ and 8.02 e^−^ in V_1O_-Ru_5_/TiO_x_ and V_2O_-Ru_5_/TiO_x_, respectively. The results obtained for the pre-oxidized structures are in good agreement with this conclusion (Supplementary Fig. 41). The electron distribution is further proved by the charge density differences (Supplementary Fig. 42), the significant charge redistributions in V_1O_-RuO/TiO_x_ indicate stronger interaction between them than P-RuO_1.6_/TiO_x_.

In addition, the introduction of V_O_ at the interface can not only enhance the antioxidant capacity of the catalyst, but also improve its performance for OER. Under acidic conditions, OER involves four proton-electron transfer steps at surface active sites, resulting in the formation of three intermediates (*OH, *O, and *OOH)^42,43^. The potential determining step (PDS) for all structural models evaluated is the third proton-electron transfer step of *O → *OOH, mainly due to the excessive adsorption of oxygen (Fig. 5c). Compared to P-RuO_1.6_/TiO_2_, the overpotential is reduced from 0.82 V to 0.70 V (V_1O_-RuO/TiO_x_) and 0.69 V (V_2O_-RuO/TiO_x_) when V_O_ is introduced at the interface. The spin-polarized total density of states (TDOS) and projected density of states (PDOS) of Ru’s *d*-orbitals for the P-RuO_1.6_/TiO_2_ and the V-RuO/TiO_x_ models are shown in Fig. 5d and Supplementary Figs. 46 and 47. The analysis of the *d*-band center results of Ru atoms at the interface shows a shift from −1.01 eV (P-RuO_1.6_/TiO_2_) to −1.33 eV (V_1O_-RuO/TiO_x_) and −1.66 eV (V_2O_-RuO/TiO_x_) upon the formation of V_O_ (Fig. 5d and Supplementary Fig. 46), indicating the weaker binding to adsorbates according to the classical *d*-band theory^44^. More importantly, the V-RuO/TiO_x_ systems have more states around Fermi level than P-RuO/TiO_x_, indicating high electrical conductivity (Supplementary Fig. 47). It is possible that V_O_ induces a stronger interaction at the interface and changes the electronic structure of the RuO/TiO_x_ system. Furthermore, Crystal orbital Hamilton population (COHP) analysis was performed to evaluate the strength of Ru-O bonds in *O intermediates (Fig. 5e). The integrated area of COHP (ICOHP) is directly proportional to the bond strength, and a higher electron orbital overlap (lower ICOHP) indicates a stronger bond^45^. Compared to P-RuO_1.6_/TiO_2_, V_1O_-RuO/TiO_x_ exhibits increased occupancy of the anti-bonding state, resulting in a decrease in the -COHP of *O from 8.23 to 7.50, indicating a stronger Ru-O bond (Supplementary Table 12). Therefore, these results demonstrate the beneficial effect of introducing V_O_ in maintaining the low valence state of the Ru active site and optimizing the binding energies of key intermediates, thereby significantly enhancing the activity and stability of OER.

## Discussion

In summary, we have proposed a one-step synthesis strategy by utilizing the redox reaction between Ru ions and Ti substrate to construct non-stoichiometric TiO_x_ supported Ru NPs as a binder-free electrode towards OER in acidic media. The as-prepared Ru/TiO_x_ catalyst demonstrates both record high activity and high stability: the overpotentials are only 174 and 209 mV to achieve current densities of 10 mA cm^−2^ and 100 mA cm^−2^, respectively, with a stability for 900 h under 10 mA cm^−2^. This enhanced performance also allows the Ru/TiO_x_ to work in pure natural seawater electrolysis. Both experimental results and DFT calculations revealed the crucial role of in situ non-stoichiometric oxides, which induced structural confinement and charge accumulation at Ru sites, preventing the aggregation and over-oxidation of Ru, thereby maintaining the OER performance during the long-term stability test. The concepts of in situ metal-support interaction and fabrication of nonstoichiometric compounds not only offer paths to the next-generation OER catalysts but also illustrate a promising way to design active sites for nanocatalysts across the wide range of conceivable systems.

## Methods

### Synthesis of Ru/TiO_x_ samples

Ru/TiO_x_ self-supporting electrode was prepared through a one-step hydrothermal method. Prior to the synthesis, a piece of Ti foam (1.0 × 6.0 cm, thickness of 0.6 mm) was sonicated in distilled water, acetone, and ethanol sequentially to remove surface oil stains and oxide layer, followed by etching the TF in the solution of HCl (18 wt%) at 363 K for 15 min. In a typical procedure, a certain amount of RuCl_3_·3H_2_O was dissolved in deionized water (10 mmol·L^−1^) with HCl (3 wt%) under vigorous stirring to form a uniform solution. The resulting solution was transferred into a 50 mL Teflon-lined stainless-steel autoclave with a piece of etched TF immersed into the reaction solution. The autoclave was sealed and heated to 200 °C for 20 h, and then cooled to room temperature. The obtained material was washed three times with deionized water in ultrasound (10 s each) and then treated by vacuum drying at 60 °C overnight.

### Synthesis of control samples

For comparison, an in situ formed bare TiO_2_/TF was synthesized using the etched-TF via the same method without the addition of RuCl_3_·3H_2_O. The obtained Ru/TiO_x_ was further annealed in air at 450 °C for 1 h to prepare annealed RuO_x_/TiO_2_. The benchmark IrO_2_ and RuO_2_ catalysts on Ti foam were fabricated by the following steps: 280 μL ethanol, 20 μL Nafion, 70 μL deionized water, and 50 mg IrO_2_/RuO_2_ (Alfa Aesar) were mixed to prepare a dispersion, then add the prepared dispersion to the TF (the mass added to the electrode is determined according to the loading mass).

### Material characterizations

The morphology, energy dispersion spectra (EDS), and elemental mapping of the samples were collected by a scanning electron microscope (FE-SEM, JSM-7500, Japan), transmission electron microscope (FE-TEM, G2F20, USA) and scanning transmission electron microscopy (STEM, Tecnai G2F30). Powder X-ray diffraction (XRD) patterns were collected using a Rigaku Smartlab diffractometer with Cu Kα radiation (λ = 1.5418 Å). The elements and corresponding valence states were analyzed by X-ray photoelectron spectroscopy (XPS) (ESCLAB 250Xi, Thermo Fisher Scientific). Aberration-corrected high angle annular dark field-scanning TEM (HAADF-STEM) images were collected using a Titan Cubed Themis G2 300 Double Aberration-Corrected Transmission Electron Microscope operating at 300 kV. The liquid and bubble contact angles were measured by the captive bubble method at ambient temperature (Dataphysics-OCA50, German). The accurate contents of Ru element in electrolytes were characterized by the inductively coupled plasma mass spectrometry (ICP-MS) measurement (iCAP Q, Thermo, Waltham, USA). The synchrotron-based X-ray absorption fine structure (XAFS) measurements were performed with Si (311) crystal monochromators at the BL14W1 beamlines at the Beijing Synchrotron Radiation Facility (BSRF) (Beijing, China). The XAFS spectra were recorded at room temperature using a 4-channel Silicon Drift Detector (SDD) Bruker 5040. Ru *K*-edge extended X-ray absorption fine structure (EXAFS) spectra were recorded in transmission mode. Negligible changes in the line-shape and peak position of Ru K-edge XANES spectra were observed between two scans taken for a specific sample. The XAFS spectra of the standard samples (Ru foil and RuO_2_) were recorded in transmission mode. The spectra were processed and analyzed by the software codes Athena and Artemis. In-situ XPS spectra were measured by ambient pressure XPS end station equipped with a static electrochemical cell at ESCLAB 250Xi (Supplementary Fig. 27). The counter electrode was Pt and the reference electrode was saturated calomel electrode (SCE). The potentials of the Ru/TiO_x_ as a working electrode (1.0−1.7 V vs. RHE) were precisely controlled.

### Electrochemical measurements

Electrochemical measurements were performed with a workstation in a typical three-electrode configuration consisting of a Pt plate (the counter electrode), an Ag/AgCl electrode (the reference electrode), and the active material (the working electrode) in 0.5 M H_2_SO_4_ solution (pH = 0.3). The pH of the electrolyte was measured using a pH-meter (Mettler Toledo, Germany). The linear sweep voltammetry (LSV) polarization curves were collected at a scan rate of 5 mV s^−1^. Therein, all of the measured potentials versus the reference electrode were converted to a reversible hydrogen electrode (RHE) according to the equation (E_RHE_ = E_Ag/AgCl_ + 0.197 V +  0.0591 × pH). The overpotential values reported in the manuscript are all obtained through the LSV curves without *iR* correction. For comparison, the LSV curves with 95 % *iR* (*i*, current; *R*, resistance) compensation were also reported. The OER stability was evaluated by continuous cyclic voltammograms (CVs) up to 550 mA cm^−2^ for 50 cycles and chronopotentiometry test at constant current densities of 10 mA cm^−2^ and 100 mA cm^−2^. The chronopotentiometric tests of the samples under a constant OER current density of 10 and 100 mA cm^−2^ were conducted in an H-type water electrolysis cell with the anode and cathode separated by a Nafion 117 membrane. The LSV polarization curves and chronopotentiometric results were obtained under the same operation conditions in natural seawater (Yellow Sea, 120°E, 35°N) without any further treatment. Nyquist plots of electrochemical impedance spectroscopy (EIS) measurements were collected in the frequency range of 100 kHz to 0.01 Hz at open-circuit potential with an amplitude of 5 mV AC voltage in 0.5 M H_2_SO_4_ solution. The electrochemical accessible surface area (ECSA) was determined by: ECSA = *C*_dl_/*C*_s_, where *C*_dl_ is double-layer capacitance and *C*_s_ the specific capacitance of the sample. In this work, a general specific capacitance of *C*_s_  =  0.035 mF cm^−2^ was used based on typically reported values^3.28^. *C*_dl_ was determined by the equation *C*_dl_  =  *i*_c_/*ν*, where *i*_c_ is the charging current and *v* is the scan rate. A series of CV tests in the non-faradaic potential region (0.2 ~ 0.3 V vs. RHE) with various scan rates (20, 40, 60, 80, 100, 120 mV s^−1^) were performed. By plotting measured *i*_c_ versus *ν*, *C*_dl_ was obtained from the slopes of the linear fitting. Typically, the pH-dependence measurement was carried out at 1.23 to 1.53 V vs. RHE in H_2_SO_4_ with different pH (0.3, 0.4, 0.7, and 1). For electrolyser tests, a self-made cell was used as the PEMWE device and a cation exchange membrane (Nafion 212) as the membrane electrolyte. The membrane electrode assembly (MEA) was prepared by pressing the cathodes (20% Pt/C sprayed on the Nafions 212 membrane) and anodes (self-supported Ru/TiO_x_). During the test, the cell was maintained at 60°C, and the pre-heated deionized water was fed to the anode by a peristaltic pump. All the data of PEMWE were not *iR* corrected and displayed as raw data.

### Computational details

Density functional theory (DFT) calculations^46,47^, were performed as implemented in the Vienna ab initio simulation package (VASP 5.4.4)^48,49^ in conjunction with VASPsol^50,51^. The projected augmented wave (PAW) potential and the generalized gradient approximation (GGA) with the Perdew-Burke-Ernzerhof (PBE) functional were used to describe the electron-ion interactions and the exchange-correlation energy, respectively. The cutoff energy for the plane-wave basis set was set to 500 eV. A force tolerance was set as 0.02 eV Å^−1^ on each atom for structural relaxation. A 2 × 3 × 1 Monkhorst-Pack *k*-point sampling was used for geometry optimization, and a 3 × 4 × 1 Monkhorst-Pack *k*-point sampling in the Brillouin zone was used for electronic structure calculation. A Hubbard correction of *U* = 2.5 eV, *J* = 0 eV, and *U* = 3.0 eV, *J* = 0.5 eV was applied to Ti and Ru atoms. The (110) surface of TiO_2_ was simulated as a four-layer slab with ~15 Å vacuum layer along the c-axis, where the bottom TiO_2_ were fixed at their optimal bulk positions and the remaining atoms were fully relaxed.

In this work, we considered OER to occur as the following four steps:

1 $$*+{{{{{{\rm{OH}}}}}}}^{-}\to*{{{{{\rm{OH}}}}}}+{{{{{{\rm{e}}}}}}}^{-}$$

2 $$*{{{{{\rm{OH}}}}}}+{{{{{{\rm{OH}}}}}}}^{-}\to*{{{{{\rm{O}}}}}}+{{{{{{\rm{H}}}}}}}_{2}{{{{{\rm{O}}}}}}+{{{{{{\rm{e}}}}}}}^{-}$$

3 $$*{{{{{\rm{O}}}}}}+{{{{{{\rm{OH}}}}}}}^{-}\to*{{{{{\rm{OOH}}}}}}+{{{{{{\rm{e}}}}}}}^{-}$$

4 $$*{{{{{\rm{OOH}}}}}}+{{{{{{\rm{OH}}}}}}}^{-}\to*+{{{{{{\rm{O}}}}}}}_{2}({{{{{\rm{g}}}}}})+{{{{{{\rm{H}}}}}}}_{2}{{{{{\rm{O}}}}}}+{{{{{{\rm{e}}}}}}}^{-}$$

where * is the adsorption site for the intermediates. For each step of OER, the free energy of reaction $\Delta G$ was studied within the framework of the computational hydrogen electrode^43,52^, where ${H}_{2}$ is in equilibrium with both protons and electrons:

5 $$\frac{1}{2}{{{{{{\rm{H}}}}}}}_{2}\to {{{{{{\rm{H}}}}}}}^{+}+{{{{{{\rm{e}}}}}}}^{-}$$

Then $\Delta G$ was calculated by the following equation:

6 $$\Delta G=\Delta E+\Delta {ZPE}-T\Delta S+\Delta {G}_{U}$$

where $\Delta E$ is the DFT energy difference of the reactions, $\Delta {ZPE}$ is the zero-point energy correction, $\Delta S$ is the vibrational entropy change at 298.15 K, $\Delta {G}_{U}=-{eU}$, *U* = 1.23 V.

The overpotential *η* of OER can be evaluated from the largest $\Delta G$ of each step as:

7 $${\eta }_{{OER}}=\frac{\max \left\{\Delta {G}_{1},\, \Delta {G}_{2},\, \Delta {G}_{3},\, \Delta {G}_{4}\right\}}{e}$$

where $\Delta {G}_{1},\, \Delta {G}_{2},\, \Delta {G}_{3},{{{{{\rm{and}}}}}}\,\Delta {G}_{4}$ are the free energy of reactions (1) to (4), respectively.

Ru NP is simulated by Ru_5_ clusters with the configuration of the tetragonal cone, and non-stoichiometric oxide TiO_x_ is simulated by TiO_2_ (110) with O vacancy (V_O_) on the surface (V-Ru_5_/TiO_x_), while Ru_5_ clusters adsorbed on the pristine TiO_2_ (110) (P-Ru_5_/TiO_2_) were also investigated for comparison. Considering that V_O_ could enhance the antioxidant capacity of Ru_5_ cluster, P-RuO_1.6_/TiO_2_ and V-RuO/TiO_x_ were constructed to investigate the OER mechanism in the catalytic performance calculations. Detailed structural information is presented in the supplement materials. To obtain a stable TiO_x_ surface, we carried out ab initio molecular dynamics (AIMD). The AIMD simulations at 300 K for 10 ps with a time step 1 fs. The Charge density differences ($\Delta \rho={\rho }_{{{{{{\rm{RuO}}}}}}/{{{{{\rm{TiOx}}}}}}}-{\rho }_{{{{{{\rm{RuO}}}}}}}-{\rho }_{{{{{{\rm{TiOx}}}}}}}$) are calculated to express the interaction between RuO and TiO_x_. For V-RuO/TiO_x_, the significant charge redistributions indicate a stronger interaction between them than P-RuO/TiO_x_.

### Supplementary information

Supplementary Information

Peer review file

Description of additional supplementary files

Supplementary Movie 1

Supplementary Movie 2

## Data Availability

The data that support the findings of this study are presented in the main text and the Supplementary Information, and are available from the corresponding authors upon reasonable request. The Source Data underlying the figures of this study are available at 10.6084/m9.figshare.24324541. All raw data generated during the current study are available from the corresponding authors upon request.

## References

[1] Nong HN (2020). Key role of chemistry versus bias in electrocatalytic oxygen evolution. Nature.

[2] Pomerantseva E, Bonaccorso F, Feng X-L, Cui Y, Gogotsi Y (2019). Energy storage: the future enabled by nanomaterials. Science.

[3] Wu Z-Y (2023). Non-iridium-based electrocatalyst for durable acidic oxygen evolution reaction in proton exchange membrane water electrolysis. Nat. Mater..

[4] Yao Y-C (2019). Engineering the electronic structure of single atom Ru sites via compressive strain boosts acidic water oxidation electrocatalysis. Nat. Catal..

[5] Li J (2021). Molecular and heterogeneous water oxidation catalysts: recent progress and joint perspectives. Chem. Soc. Rev..

[6] Du K (2022). Interface engineering breaks both stability and activity limits of RuO_2_ for sustainable water oxidation. Nat. Commun..

[7] Lin C (2021). In-situ reconstructed Ru atom array on α-MnO_2_ with enhanced performance for acidic water oxidation. Nat. Catal..

[8] Kuhn AT, Chan C-Y (1983). pH changes at near-electrode surfaces. J. Appl. Electrochem.

[9] Lu X-Y (2018). A sea-change: manganese doped nickel/nickel oxide electrocatalysts for hydrogen generation from seawater. Energy Environ. Sci..

[10] Lin Y-C, Dong Y, Wang X-Z, Chen L (2022). Electrocatalysts for the oxygen evolution reaction in acidic media. Adv. Mater..

[11] An L (2021). Recent development of oxygen evolution electrocatalysts in acidic environment. Adv. Mater..

[12] Cui X-J (2020). Robust interface Ru centers for high-performance acidic oxygen evolution. Adv. Mater..

[13] Cao L-L (2019). Dynamic oxygen adsorption on single-atomic ruthenium catalyst with high performance for acidic oxygen evolution reaction. Nat. Commun..

[14] Anantharaj S, Noda S, Driess M, Menezes PW (2021). The pitfalls of using potentiodynamic polarization curves for Tafel analysis in electrocatalytic water splitting. ACS Energy Lett..

[15] Li L (2021). Compensating electronic effect enables fast site-to-site electron transfer over ultrathin RuMn nanosheet branches toward highly electroactive and stable water splitting. Adv. Mater..

[16] Wu D-S (2021). Efficient overall water splitting in acid with anisotropic metal nanosheets. Nat. Commun..

[17] Qin Y (2022). RuO_2_ electronic structure and lattice strain dual engineering for enhanced acidic oxygen evolution reaction performance. Nat. Commun..

[18] Zhang L-J (2021). Sodium-decorated amorphous/crystalline RuO_2_ with rich oxygen vacancies: a robust pH-universal oxygen evolution electrocatalyst. Angew. Chem., Int. Ed..

[19] Wang K-X (2022). Highly active ruthenium sites stabilized by modulating electron-feeding for sustainable acidic oxygen-evolution electrocatalysis. Energy Environ. Sci..

[20] Chandrasekaran S (2022). Developments and perspectives on robust nano- and microstructured binder-free electrodes for bifunctional water electrolysis and beyond. Adv. Energy Mater..

[21] Yang L-P (2022). Atomic Fe–N_4_/C in flexible carbon fiber membrane as binder-free air cathode for Zn-Air batteries with stable cycling over 1000 h. Adv. Mater..

[22] Ye M-D, Liu HY, Lin C-J, Lin Z-Q (2013). Hierarchical rutile TiO_2_ flower cluster-based high efficiency dye-sensitized solar cells via direct hydrothermal growth on conducting substrates. Small.

[23] Allam NK, Grimes CA (2007). Formation of vertically oriented TiO_2_ nanotube arrays using a fluoride free HCl. aqueous electrolyte J. Phys. Chem. C..

[24] Xie Y (2009). In situ controllable loading of ultrafine noble metal particles on titania. J. Am. Chem. Soc..

[25] Huang X-P, Pan C-X (2007). Large-scale synthesis of single-crystalline rutile TiO_2_ nanorods via a one-step solution route. J. Cryst. Growth.

[26] Liang S-Y, Zheng X, Zhu J, Yu R (2018). Coherent topotactic interface between corundum and rutile Structures. J. Phys. Chem. C..

[27] Tsujimoto Y, Matsushita Y, Yu S, Yamaura K, Uchikoshi T (2018). Size dependence of structural, magnetic, and electrical properties in corundum-type Ti_2_O_3_ nanoparticles showing insulator-metal transition. J. Asian Ceram. Soc..

[28] Li Y-Y (2019). Electronic-reconstruction-enhanced hydrogen evolution catalysis in oxide polymorphs. Nat. Commun..

[29] Rong C-L (2022). Electronic structure engineering of single-atom Ru Sites via Co-N_4_ Sites for bifunctional pH-universal water splitting. Adv. Mater..

[30] Yao Q (2021). A chemical etching strategy to improve and stabilize RuO_2_-based nanoassemblies for acidic oxygen evolution. Nano Energy.

[31] Shan J-Q (2019). Charge-redistribution-enhanced nanocrystalline Ru@IrO_x_ electrocatalysts for oxygen evolution in acidic media. Chem.

[32] Xu YL, Wang C, Huang Y-H, Fu J (2021). Recent advances in electrocatalysts for neutral and large-current-density water electrolysis. Nano Energy.

[33] Dresp S, Dionigi F, Klingenhof M, Strasser P (2019). Direct electrolytic splitting of seawater: opportunities and challenges. ACS Energy Lett..

[34] Dresp S (2018). Direct electrolytic splitting of seawater: activity, selectivity, degradation, and recovery studied from the molecular catalyst structure to the electrolyzer cell level. Adv. Energy Mater..

[35] Yu L (2019). Non-noble metal-nitride based electrocatalysts for high-performance alkaline seawater electrolysis. Nat. Commun..

[36] Zhao Y-Q (2018). Charge state manipulation of cobalt selenide catalyst for overall seawater electrolysis. Adv. Energy Mater..

[37] Li X-Y (2020). Controlling CO_2_ hydrogenation selectivity by metal-supported electron transfer. Angew. Chem., Int. Ed..

[38] Zhou Y-Y (2020). Lattice-confined Ru clusters with high CO tolerance and activity for the hydrogen oxidation reaction. Nat. Catal..

[39] Wang Y (2023). Unraveling oxygen vacancy site mechanism of Rh-doped RuO_2_ catalyst for long-lasting acidic water oxidation. Nat. Commun..

[40] Wang J (2022). Exceptionally active and stable RuO_2_ with interstitial carbon for water oxidation in acid. Chem.

[41] Seitz LC (2016). A highly active and stable IrO_x_/SrIrO_3_ catalyst for the oxygen evolution reaction. Science.

[42] Gou, W. Y. et al. Highly active and stable amorphous IrO_x_/CeO_2_ nanowires for acidic oxygen evolution. *Nano Energy* **104**, 107960 (2022).

[43] Man IC (2011). Universality in oxygen evolution electrocatalysis on oxide surfaces. ChemCatChem.

[44] Jin H (2022). Safeguarding the RuO_2_ phase against lattice oxygen oxidation during acidic water electrooxidation. Energy Environ. Sci..

[45] Jiao JY (2022). Tuning of surface morphology in Li layered oxide cathode materials. Acta Mater..

[46] Hohenberg P, Kohn W (1964). Density functional theory (DFT). Phys. Rev..

[47] Kohn W, Sham LJ (1965). Self-consistent equations including exchange and correlation effects. Phys. Rev..

[48] Kresse G, Hafner J (1993). Ab initio molecular dynamics for liquid metals. Phys. Rev. B.

[49] Kresse G, Furthmüller J (1996). Efficient iterative schemes for ab initio total-energy calculations using a plane-wave basis set. Phys. Rev. B.

[50] Mathew, K., Kolluru, V. S. C., Hennig, R. G. VASPsol: implicit solvation and electrolyte model for density-functional theory. https://github.com/henniggroup/VASPsol (2018).

[51] Mathew K, Sundararaman R, Letchworth-Weaver K, Arias TA, Hennig RG (2014). Implicit solvation model for density-functional study of nanocrystal surfaces and reaction pathways. J. Chem. Phys..

[52] Nørskov JK (2004). Origin of the overpotential for oxygen reduction at a fuel-cell cathode. J. Phys. Chem. B.

