# Supplementary material for: Stabilizing non-iridium active sites by non-stoichiometric oxide for acidic water oxidation at high current density
Source: Nat Commun. 2023 Nov 23;14:7644. doi: 10.1038/s41467-023-43466-x (PMC10667250; doi:10.1038/s41467-023-43466-x)
Supplement: Supplementary file 1 — Supplementary Information [file 41467_2023_43466_MOESM1_ESM.pdf]

**Supplementary Information**

# Stabilizing non-iridium active sites by non-stoichiometric oxide for acidic water oxidation at high current density

Lingxi Zhou[1,†], Yang-Fan Shao[2,†], Fang Yin[2], Jia Li[2*], Feiyu Kang[2,3] and Ruitao Lv[1,3*]

[1] State Key Laboratory of New Ceramics and Fine Processing, School of Materials Science and Engineering, Tsinghua University, Beijing 100084, China

[2] Institute of Materials Research and Shenzhen Geim Graphene Center, Tsinghua Shenzhen International Graduate School, Tsinghua University, Shenzhen 518055, China

[3] Key Laboratory of Advanced Materials (MOE), School of Materials Science and Engineering, Tsinghua University, Beijing 100084, China

[†] These authors contributed equally: Lingxi Zhou and Yang-Fan Shao.

[*] Corresponding authors. Email addresses:

li.jia@sz.tsinghua.edu.cn (J. Li)

lvruitao@tsinghua.edu.cn (R. Lv)

Contents

**Supplementary Figure 1.** Optical photo of Ti foam (TF, left) and as-prepared Ru/$TiO_x$ (right)............................6

**Supplementary Figure 2.** Morphology characterization of TF. ............................................................6

**Supplementary Figure 3.** SEM image of Ru/$TiO_x$...............................................................................7

**Supplementary Figure 4.** HRTEM image of Ru/$TiO_x$. ........................................................................7

**Supplementary Figure 5.** Phase characterization of different samples. ..............................................8

**Supplementary Figure 6.** HAADF-STEM image of Ru/$TiO_x$. ............................................................8

**Supplementary Figure 7.** SEM images of bare $TiO_2$/TF. ....................................................................9

**Supplementary Figure 8.** HAADF-TEM images.....................................................................................9

**Supplementary Figure 9.** Overpotentials comparsion after *iR* correction...........................................10

**Supplementary Figure 10.** OER activity in 0.5 M $H_2SO_4$. ..................................................................10

**Supplementary Figure 11**. ECSA measurement of different samples.. ................................................11

**Supplementary Figure 12**. Specific OER activity of different samples.. ...............................................11

**Supplementary Figure 13**. Mass activity of different samples. ............................................................12

**Supplementary Figure 14.** Turnover frequency (TOF) of different samples. .......................................12

**Supplementary Figure 15.** Tafel analysis. ............................................................................................13

**Supplementary Figure 16**. Hydrophilicity of TF and Ru/$TiO_x$. ............................................................14

**Supplementary Figure 17**. Aerophobicity of TF and Ru/$TiO_x$.. ..........................................................14

**Supplementary Figure 18.** CVs of the as-prepared Ru/$TiO_x$. .............................................................15

**Supplementary Figure 19.** Electrocatalytic OER stability in 0.5 M $H_2SO_4$.........................................15

**Supplementary Figure 20.** Ru content before and after OER stability test. ..........................................16

**Supplementary Figure 21.** PEMWE device. ..........................................................................................16

**Supplementary Figure 22.** PEMWE performance. ................................................................................17

**Supplementary Figure 23.** Electrocatalytic OER performance in natural seawater. ..............................17

**Supplementary Figure 24.** O 1*s* XPS spectra of different samples.. ....................................................18

**Supplementary Figure 25.** Ti 2*p* and Ru 3*p* XPS spectra of different samples. ....................................18

**Supplementary Figure 26.** The $Ru^{n+}/Ru^0$ ratio.. ...................................................................................19

**Supplementary Figure 27.** In-situ XPS device.. .....................................................................................19

**Supplementary Figure 28.** In-situ XPS spectra of Ru/$TiO_x$ during the OER test. ................................20

**Supplementary Figure 29.** Variation of $Ru^{n+}/Ru^0$, $Ti^{3+}/Ti^{4+}$ and $O_V/O_L$..............................................20

**Supplementary Figure 30.** Fitting of X-ray absorption results.. ...........................................................21

**Supplementary Figure 31.** SEM images of Ru/$TiO_x$ before and after OER. .........................................22

**Supplementary Figure 33.** SEM images of control samples before and after OER. 24

**Supplementary Figure 34.** TEM images of control samples before and after OER. 25

**Supplementary Figure 35.** pH-dependence experiment. 25

**Supplementary Figure 36.** Calculated Pourbaix diagrams. 26

**Supplementary Figure 37.** Molecular dynamic simulation. 26

**Supplementary Figure 38.** Theoretical calculation models of P-$TiO_2$ and V-$TiO_x$. 27

**Supplementary Figure 39.** Theoretical calculation models of P-Ru/$TiO_2$ and V-Ru/$TiO_x$. 27

**Supplementary Figure 40.** Theoretical calculation models of $RuO_x$/$TiO_x$ 28

**Supplementary Figure 41.** Bader charge analysis. 28

**Supplementary Figure 42.** Differential charge density analysis 29

**Supplementary Figure 43.** Atomic structures of $V_{1O}$-RuO/$TiO_x$ with adsorbed OER intermediates. 29

**Supplementary Figure 44.** Atomic structures of P-$RuO_{1.6}$/$TiO_2$ with adsorbed OER intermediates. 30

**Supplementary Figure 45.** Atomic structures of $V_{2O}$-RuO/$TiO_x$ with adsorbed OER intermediates. 30

**Supplementary Figure 46.** PDOS analysis. 31

**Supplementary Figure 47.** TDOS analysis. 31

**Supplementary Table 1.** Summary of electrocatalytic OER performance of the Ru/$TiO_x$ catalysts and state-of-the-art electrocatalysts in acidic media. 32

**Supplementary Table 2**. The mass loading (mg $cm^{-2}$) and weight percent ($_{wt}$%) of noble metal in different samples (by ICP-MS measurement and EDS) and atomic percent ($_{at}$%) by XPS measurement. 33

**Supplementary Table 3**. OER mass activity comparison between the as-synthesized Ru/$TiO_x$ catalyst and other reported noble metal-based electrocatalysts in acidic media. 34

**Supplementary Table 4.** TOF of Ru/$TiO_x$ with previously reported OER catalysts in acid. 35

**Supplementary Table 5.** TOF of catalysts using different normalization methods. 35

**Supplementary Table 6.** Comparison of the PEM electrolyzer performance with those previously reported. 36

**Supplementary Table 7.** Concentrations of the major constituents in natural seawater. 37

**Supplementary Table 8.** High resolution Ru 3*d* XPS peak fitting parameters of different samples before and after OER. 38

**Supplementary Table 9.** High resolution Ru 3*d*, Ti 2*p* and O 1*s* XPS peak fitting parameters of Ru/$TiO_x$ at applied potential during 1.0-1.7 V vs. RHE. 39

**Supplementary Table 10**. Summary of Ru *K*-edge adsorption energy ($E_0$) and valence states for Ru foil, $RuO_2$, Ru/$TiO_x$ before and after OER. 41

**Supplementary Table 11**. Structural parameters obtained from the curve-fitting analysis of the Ru *K*-edge EXAFS spectra. 41

**Supplementary Table 12**. Integrated COHP (ICOHP) value for adsorption *O of Ru-O in different models. ...... 41

**Supplementary References** ........................................................................................................................ 42

## Supplementary Note 1:

**Calculation of turnover frequency (TOF):** The TOF value based on inductively coupled plasma mass spectrometry (ICP-MS) results (bulk TOF) and electrochemical active surface area (ECSA) values (ECSA TOF) were calculated and compared. The Bulk TOF value was calculated by following formula:

$$\mathrm{TOF} = \frac{\#\,Total\ Oxygen\ Turn\ Overs\ per\ geometric\ area}{\#\,Active\ sites\ per\ geometric\ area} \quad (1)$$

$\#\,Total\ oxygen\ turn\ overs\ per\ geometric\ area$

$$= (j\frac{mA}{cm^2})(\frac{1C\ s^{-1}}{1000\ mA})(\frac{1\ mol\ O_2}{4\ mol\ e^-})(\frac{6.022\ \times\ 10^{23}\ O_2\ atoms}{1\ mol\ O_2})(\frac{1\ mol\ e^-}{96485.3\ C})$$

$$= 1.56\ \times 10^{15}\left(\frac{O_2\ s^{-1}}{cm^2}\right)\mathrm{per}\left(\frac{mA}{cm^2}\right) \quad (2)$$

$\#\,Active\ sites\ per\ geometric\ area$

$$= \left(\frac{mass\ loading\ of\ Ru\ \left({}^{g}/_{cm^2}\right)}{Ru\ Mw\ \left({}^{g}/_{mol}\right)}\right)\left(\frac{6.022\times 10^{23}\ Ru\ atoms}{1\ mol\ Ru}\right) \quad (3)$$

The ECSA TOF value was calculated by Equation (2) and the following formula:

$$\mathrm{TOF} = \frac{\#\,Total\ Oxygen\ Turn\ Overs\ per\ geometric\ area}{\#\,Active\ sites\ per\ real\ area} \quad (4)$$

$\#\,Active\ sites\ per\ real\ surface\ area$:

$$= \left(\frac{number\ of\ active\ sites\ /\ unit\ cell}{unit\ cell\ volume}\right)^{2/3} \quad (5)$$

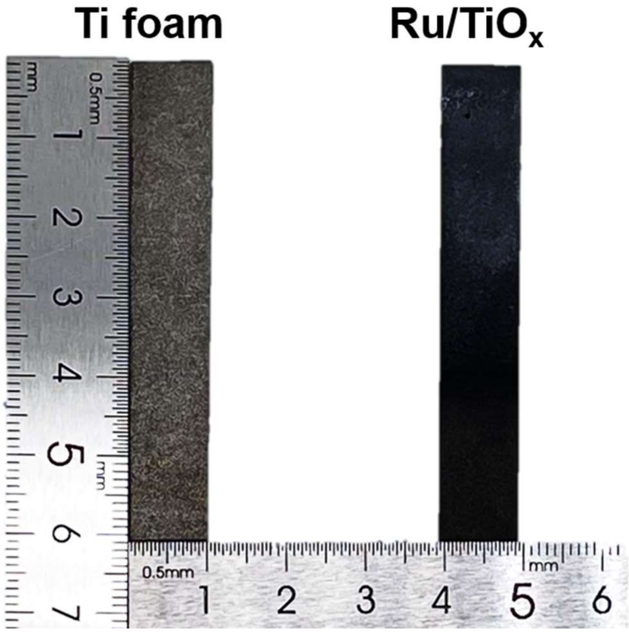


**Supplementary Figure 1.** Optical photo of Ti foam (TF, left) and as-prepared Ru/TiO$_x$ (right).

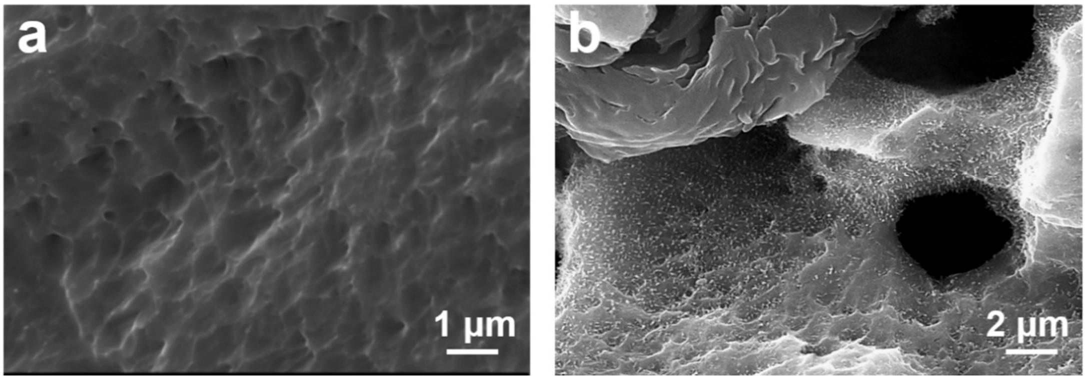


**Supplementary Figure 2. Morphology characterization of TF.** Scanning electron microscopy (SEM) image of pristine TF (**a**) and TF etched in the solution of HCl (18 wt%) at 363K for 15 min (**b**).

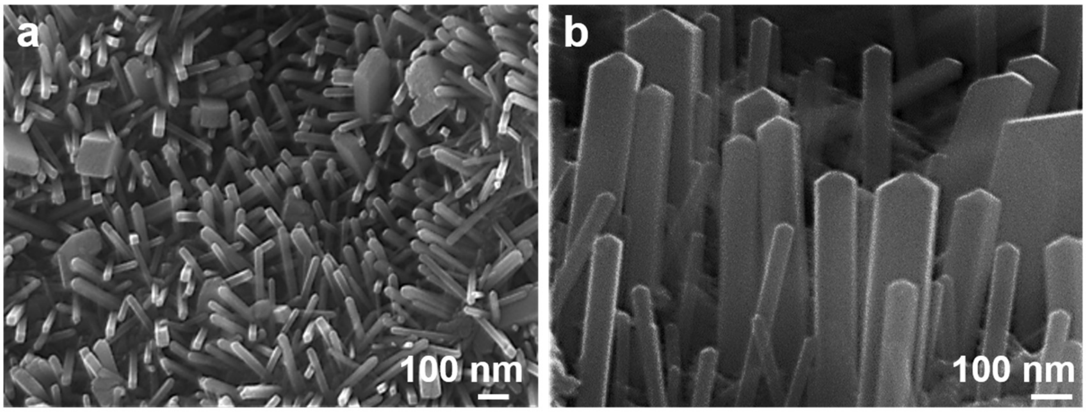


**Supplementary Figure 3.** SEM image of Ru/$TiO_x$ with top-view (**a**) and side-view (**b**).

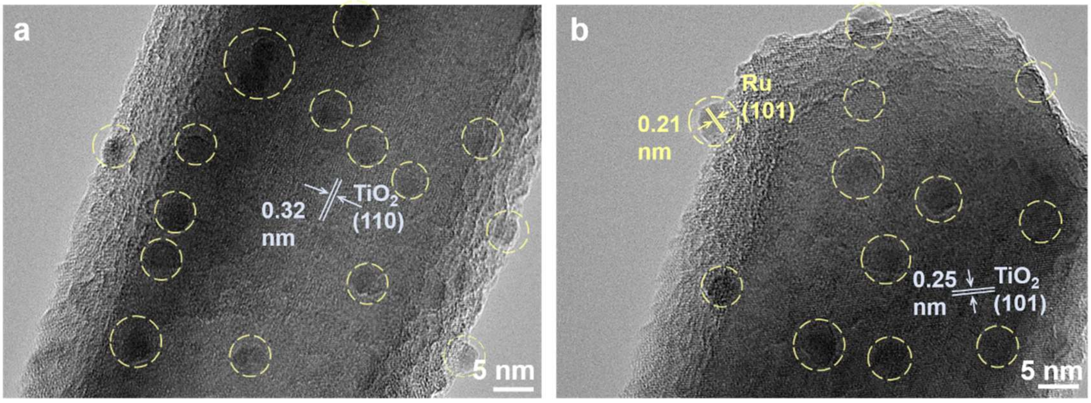


**Supplementary Figure 4.** High resolution transmission electron microscopy (HRTEM) image of Ru/$TiO_x$ with body-part (**a**) and top-part (**b**). (Ru nanoparticles are circled in yellow)

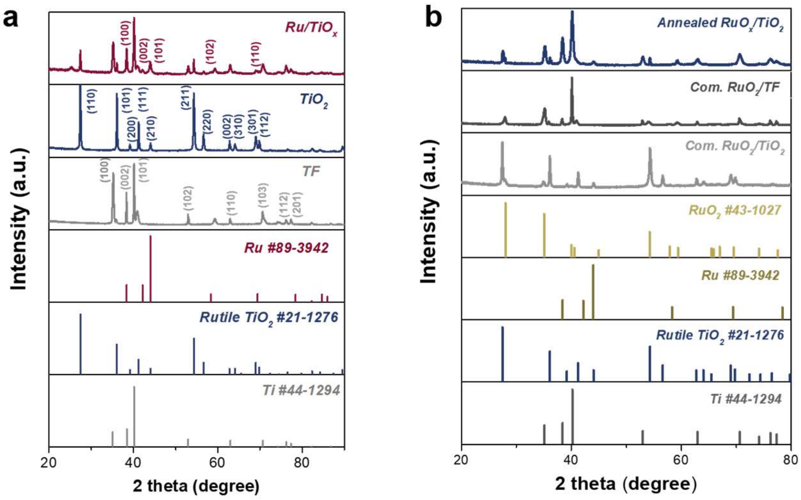


**Supplementary Figure 5. Phase characterization of different samples. a**, X-ray diffraction (XRD) patterns of Ru/$TiO_x$, $TiO_2$ and TF. **b**, XRD patterns of annealed $RuO_x$/$TiO_2$, commercial $RuO_2$/$TiO_2$ and commercial $RuO_x$/$TiO_2$ (com. denotes commercial).

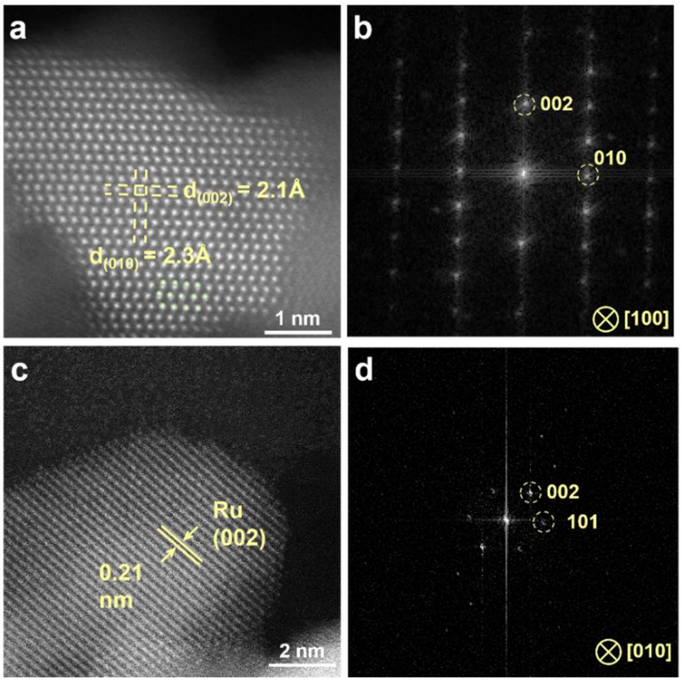


**Supplementary Figure 6.** Aberration-corrected high angle annular dark field-scanning TEM (HAADF-STEM) image of Ru/$TiO_x$ with [100] axis (**a**) and corresponding fast Fourier transforms (FFT) results (**b**). **c**, [010] axis and d) corresponding FFT results.

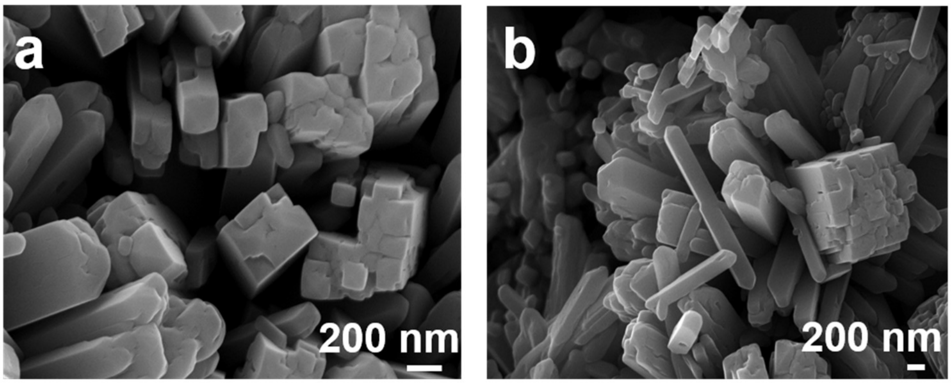


**Supplementary Figure 7.** SEM images of bare $TiO_2$/TF with different magnifications (**a**,**b**).

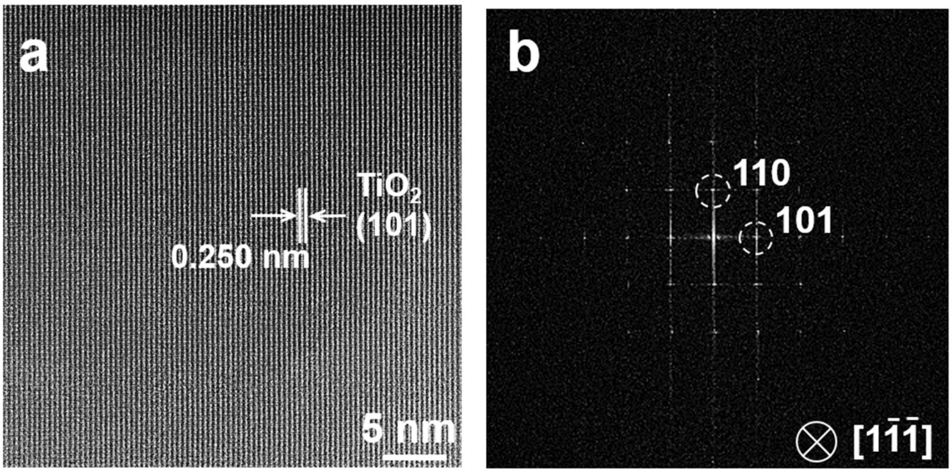


**Supplementary Figure 8.** HAADF-TEM image of bare $TiO_2$/TF (**a**) and corresponding FFT results (**b**).

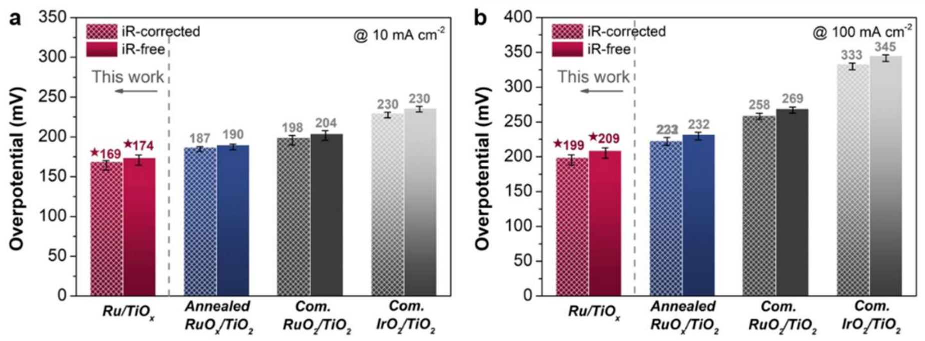


**Supplementary Figure 9.** Overpotentials of different samples to reach 10 mA cm$^{-2}$ (**a**) and 100 mA cm$^{-2}$ (**b**) before (smooth) and after (grid filled) *iR* correction. (Error bar: standard error of three repeated measurements).

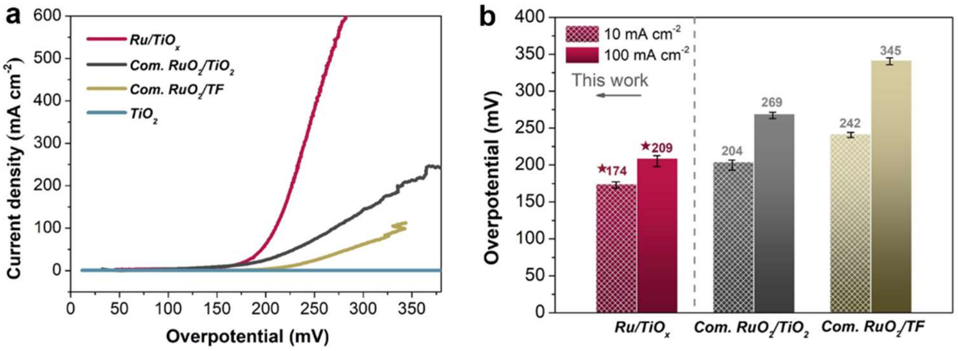


**Supplementary Figure 10. Electrocatalytic oxygen evolution reaction (OER) activity in 0.5 M $H_2SO_4$. a**, Linear sweep voltammetry (LSV) curves and **b**, comparison of overpotentials at 10 and 100 mA cm$^{-2}$ for Ru/$TiO_x$, commercial $RuO_2$/$TiO_2$, commercial $RuO_2$/TF and bare TF. Here com. denotes commercial. (Error bar: standard error of three repeated measurements).

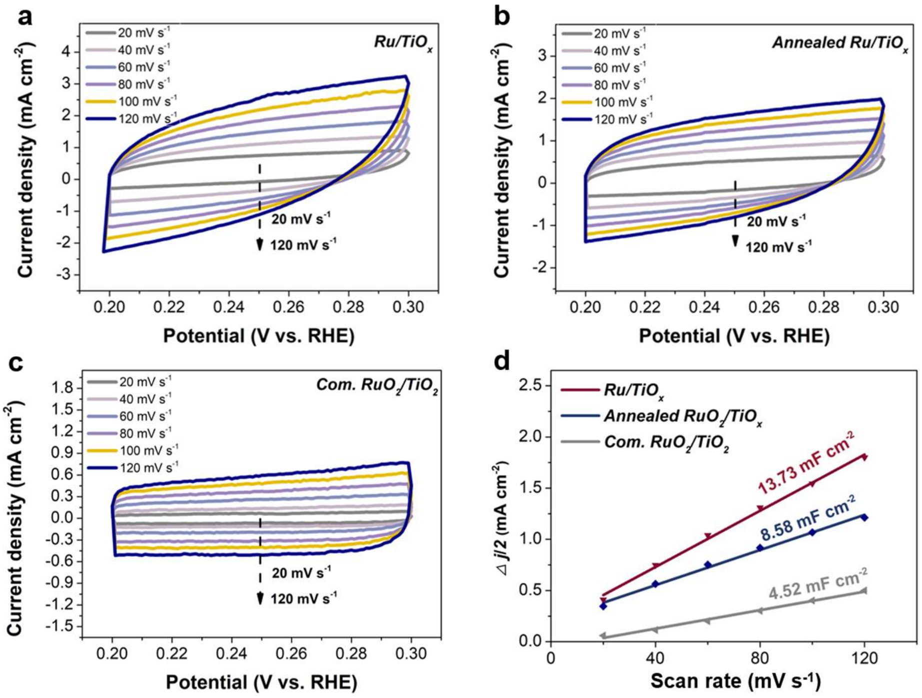


**Supplementary Figure 11**. **ECSA measurement of different samples**. **a-c**, Cyclic voltammetry (CV) curves of Ru/$TiO_x$ (**a**), annealed Ru/$TiO_x$ (**b**) and commercial $RuO_2$/$TiO_2$ (**c**). **d**, linear relationships between capacitive current and scan rate, the $C_{dl}$ are absolute value of the slope of the liner fits to the data.

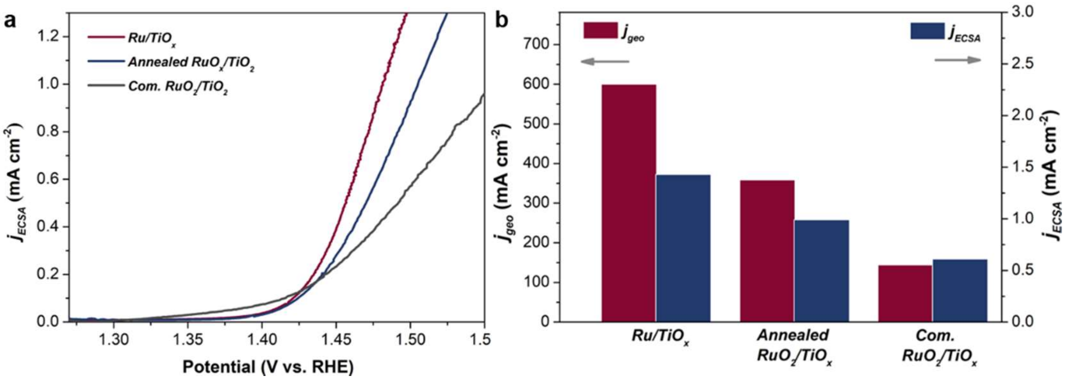


**Supplementary Figure 12**. **Specific OER activity of different samples. a**, Normalized linear sweep voltammetry curves to ECSA. **b**, The normalized OER current densities of catalysts (at 1.5 V vs. RHE) to the electrode geometrical area ($j_{geo}$) and ECSA ($j_{ECSA}$).

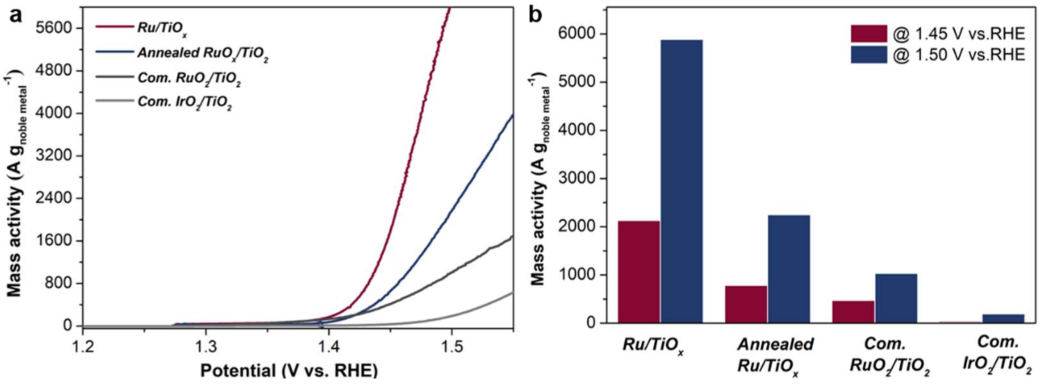


**Supplementary Figure 13**. **Mass activity of different samples**. **a**, Mass activity of Ru/TiOx, annealed $RuO_x/TiO_2$, commercial $RuO_2/TiO_2$ and commercial $IrO_2/TiO_2$ catalysts as function of applied potential in 0.5 M $H_2SO_4$. **b**, The corresponding mass activity values at potential of 1.45 and 1.50 V vs. RHE.

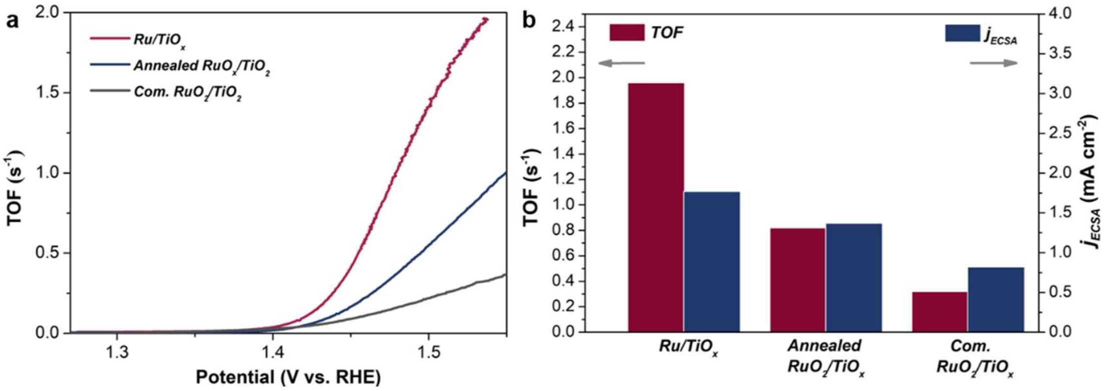


**Supplementary Figure 14. Turnover frequency (TOF) of different samples. a**, Potential-dependent TOF curves. **b**, The corresponding bar graph of the ECSA-normalized current density (blue) and TOF values (red) at the overpotential of 300 mV.

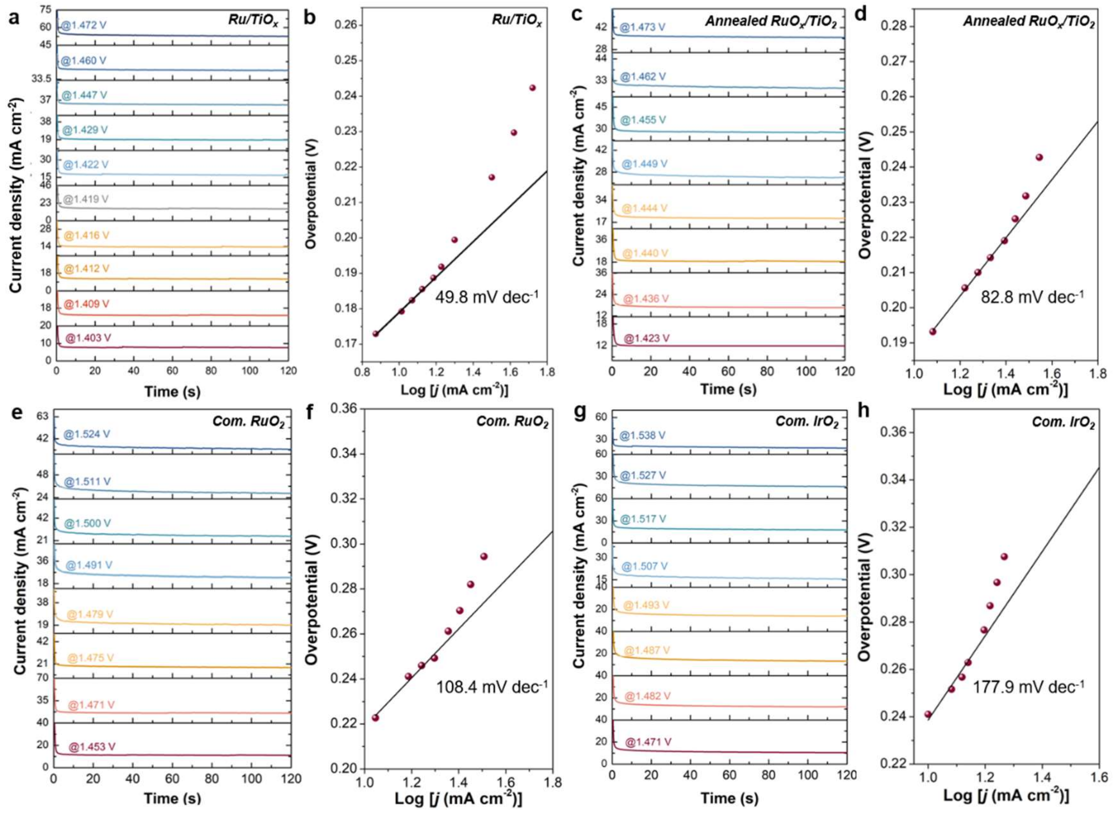


**Supplementary Figure 15.** Chronoamperometry responses of activity stabilized Ru/$TiO_x$ (**a**), annealed RuOx/$TiO_2$ (**c**), com. $RuO_2$ (**e**) and com. $IrO_2$ (**g**) in 0.5 M $H_2SO_4$. The corresponding steady-state polarization curves (Tafel plots) of Ru/$TiO_x$ (**b**), annealed RuOx/$TiO_2$ (**d**), com. $RuO_2$ (**f**) and com. $IrO_2$ (**h**) constructed from OER current densities sampled from steady-state chronoamperometry responses.

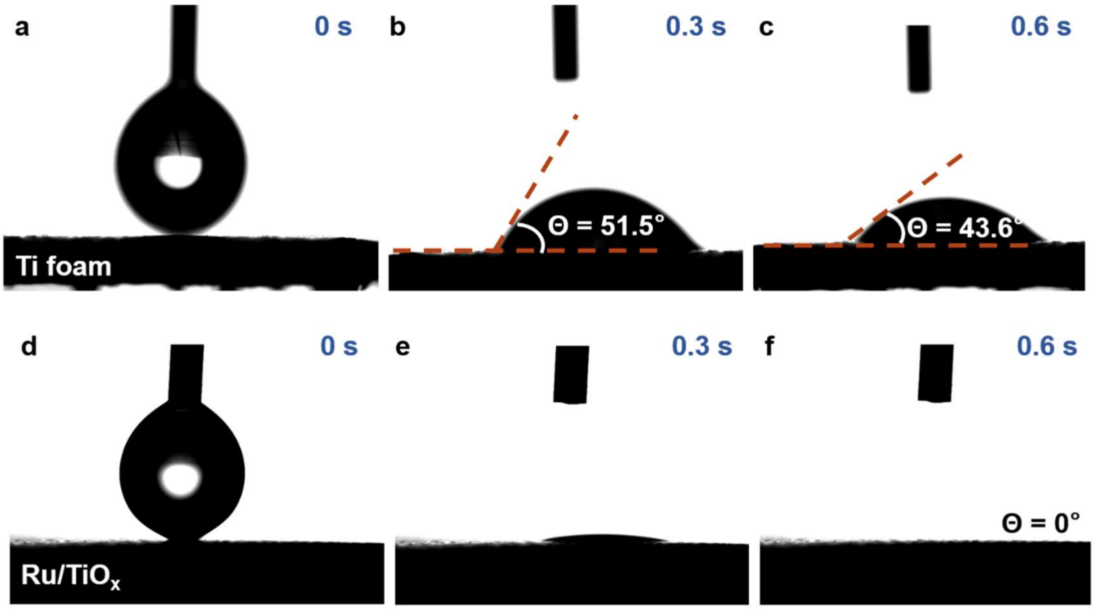


**Supplementary Figure 16**. **Hydrophilicity of TF and Ru/TiO$_x$**. The contact angles of the water droplets on TF (**a-c**) and Ru/TiO$_x$ (**d-f**).

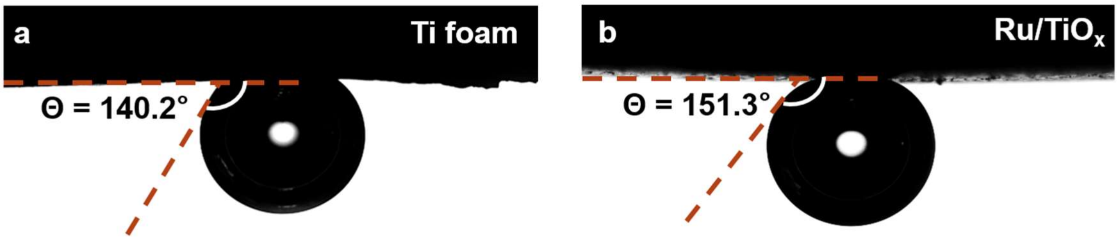


**Supplementary Figure 17**. **Aerophobicity of TF and Ru/TiO$_x$**. The bubble contact angles on the surfaces of TF (**a**) and Ru/TiO$_x$ (**b**).

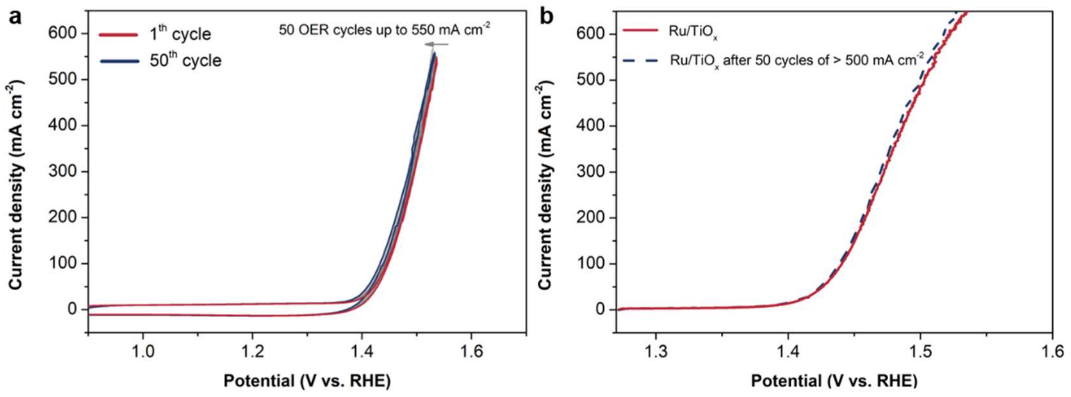


**Supplementary Figure 18.** CVs of the as-prepared Ru/$TiO_x$ up to 550 mA $cm^{-2}$ for 50 cycles (**a**) and polarization curves of Ru/$TiO_x$ before and after 50 CV cycles (**b**).

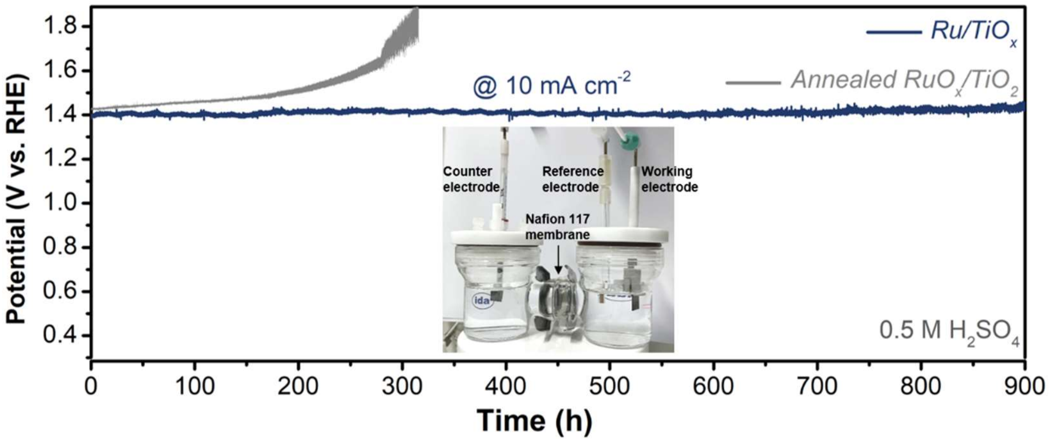


**Supplementary Figure 19. Electrocatalytic OER stability in 0.5 M $H_2SO_4$.** Chronoamperometric curve obtained at a current density of 10 mA $cm^{-2}$ for the as-prepared Ru/$TiO_x$ and the annealed $RuO_x/TiO_2$ in 0.5 M $H_2SO_4$. A photograph of a homemade H-type cell is shown in the inset, in which the anode and cathode sides are separated by a Nafion 117 membrane.

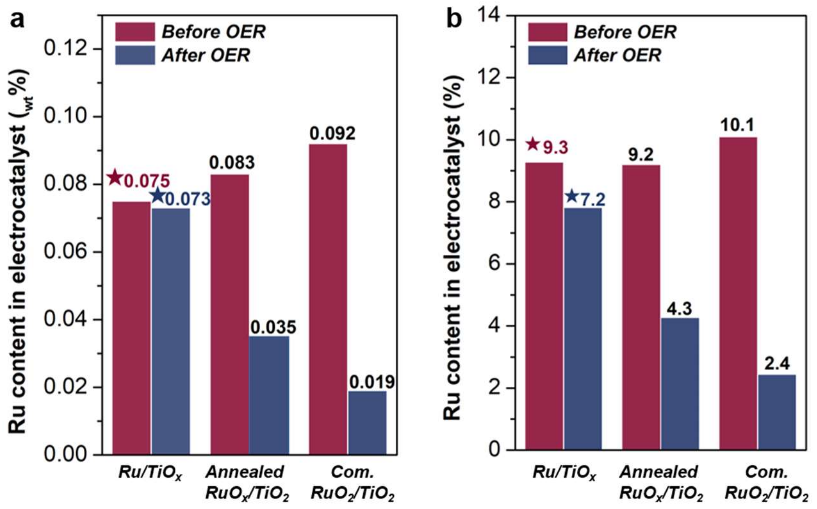


**Supplementary Figure 20. Ru content of different electrocatalyst before and after OER stability test.** Ru content in electrocatalyst before and after OER stability test determined by inductively coupled plasma-mass spectrometry (ICP-MS) (**a**) and X-ray photoelectron spectroscopy (XPS) (**b**).

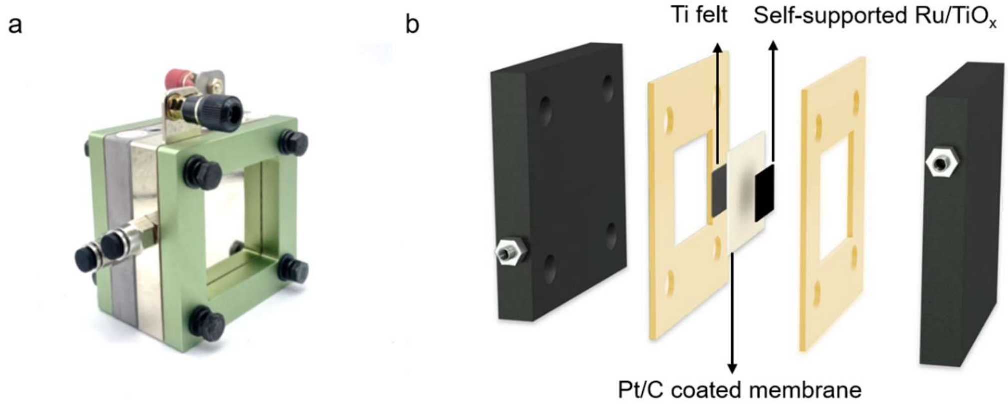


**Supplementary Figure 21.** Optical photo (**a**) and schematic diagram (**b**) of the proton-exchange membrane water electrolyzers (PEMWE).

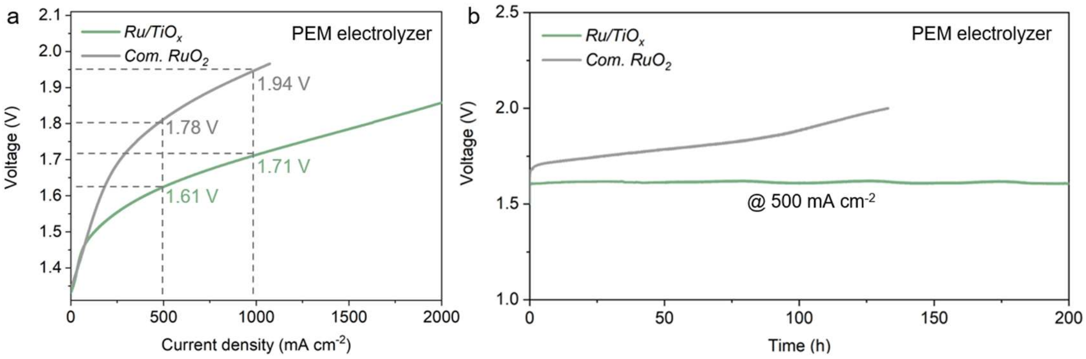


**Supplementary Figure 22. a,** Polarization curves of PEMWE utilizing the as-synthesized Ru/$TiO_x$ or commercial $RuO_2$ as an anode and commercial Pt/C as a cathode. **b**, The corresponding stability test of the PEMWE at 500 mA $cm^{-2}$.

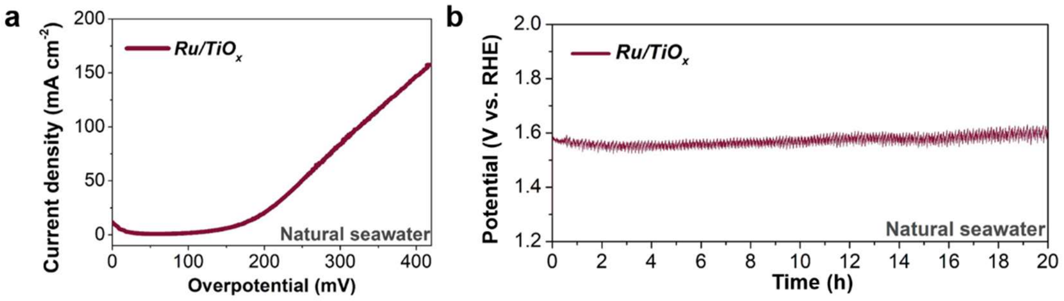


**Supplementary Figure 23. Electrocatalytic OER performance in natural seawater**. LSV curve (**a**) and chronoamperometric curve obtained at a current density of 100 mA $cm^{-2}$ for Ru/$TiO_x$ in natural seawater (**b**).

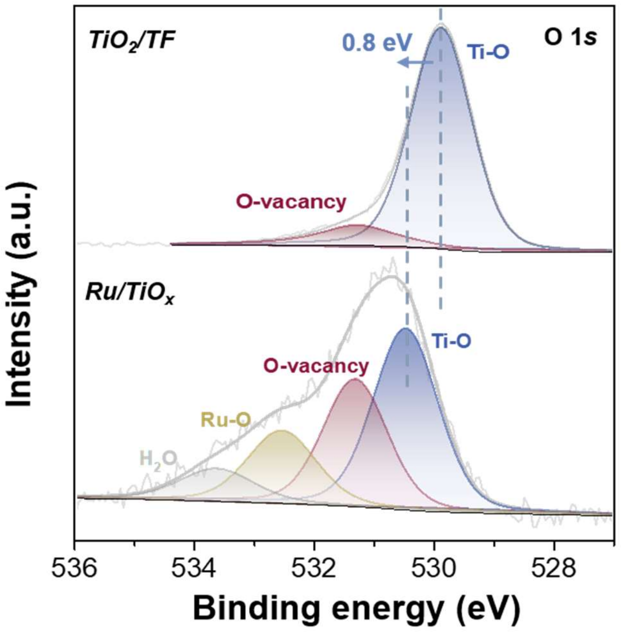


**Supplementary Figure 24. O 1*s* XPS spectra of different samples**. XPS spectra of $TiO_2$/TF (up) and Ru/$TiO_x$ (down).

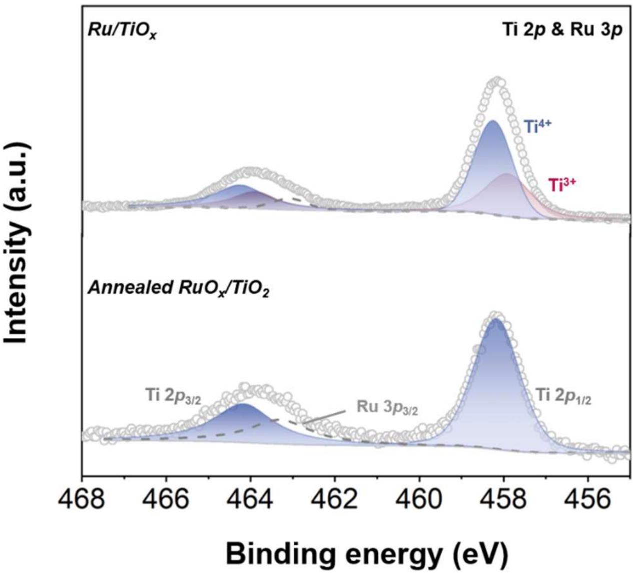


**Supplementary Figure 25. Ti 2*p* and Ru 3*p* XPS spectra of different samples**. High-resolution Ti 2*p* and Ru 3*p* XPS image of Ru/$TiO_x$ (up) and annealed $RuO_x$/$TiO_2$ (down).

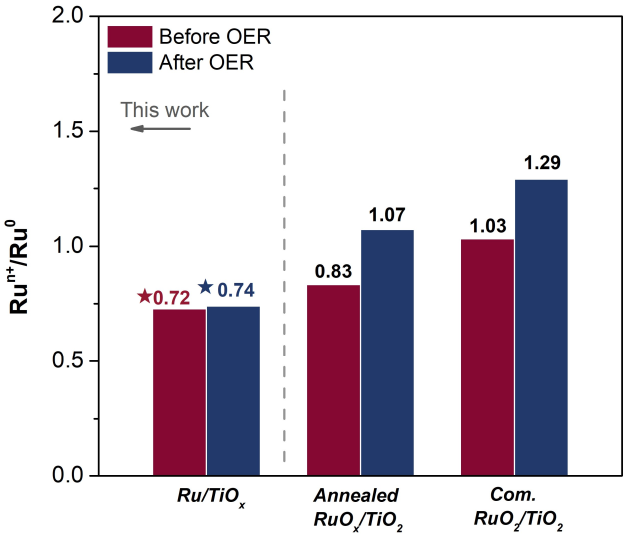


**Supplementary Figure 26.** The $Ru^{n+}/Ru^0$ ratio obtained for Ru/$TiO_x$, annealed $RuO_x/TiO_2$ and commercial $RuO_2/TiO_2$.

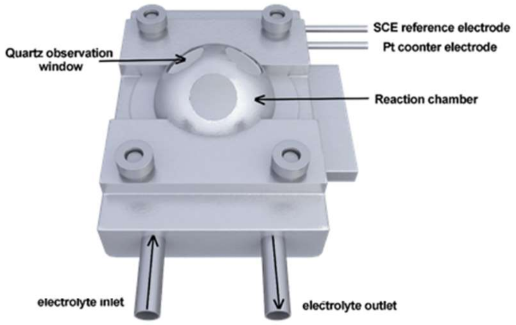


**Supplementary Figure 27.** The schematic diagram of the in-situ XPS analysis of Ru/$TiO_x$.

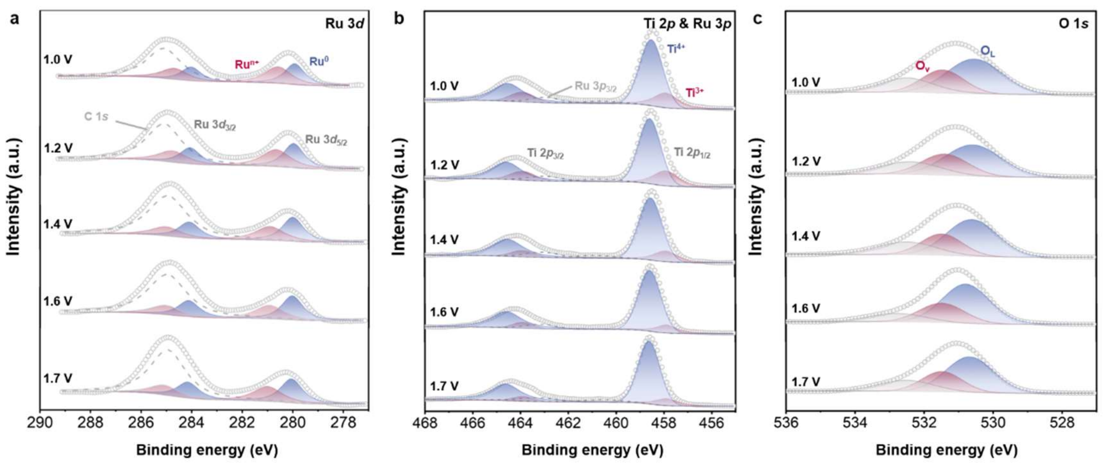


**Supplementary Figure 28. In-situ XPS spectra of Ru/TiO$_x$ during the OER test.** In-situ Ru 3*d* (**a**), Ti 2*p* & Ru 3*p* (**b**) and O 1*s* (**c**) XPS spectra recorded of the as-prepared Ru/TiO$_x$ at applied potential during 1.0-1.7 V vs. RHE.

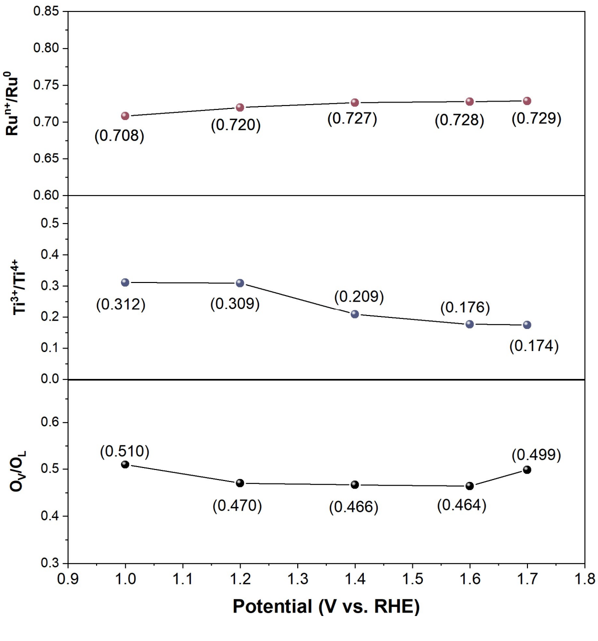


**Supplementary Figure 29.** Variation of $Ru^{n+}/Ru^{0}$, $Ti^{3+}/Ti^{4+}$ and $O_V/O_L$ (oxygen vacancy/lattice oxygen) ratio from in-situ XPS measurement.

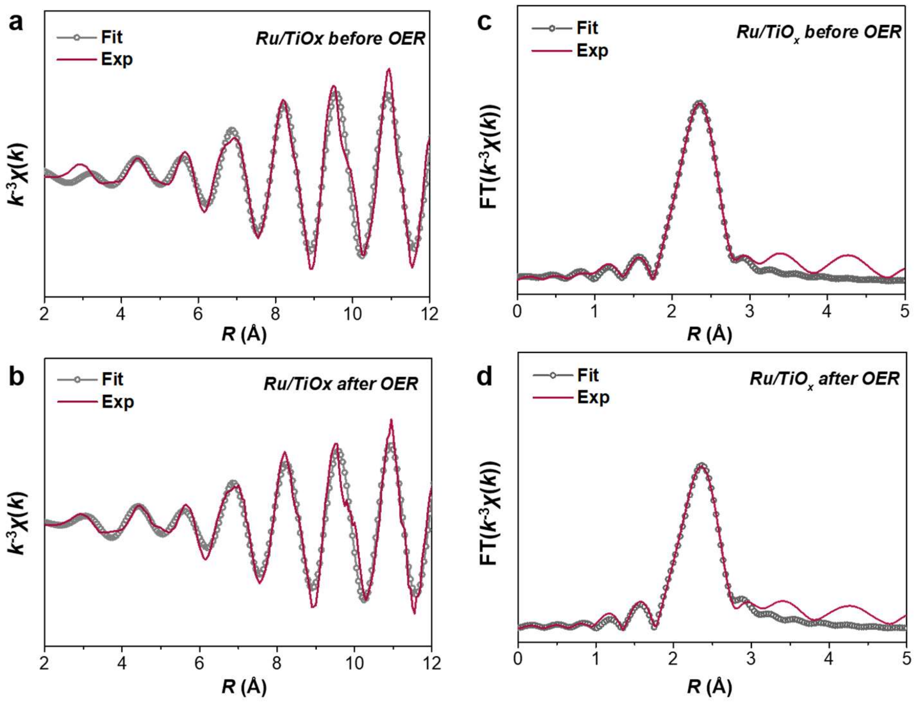


**Supplementary Figure 30. Fitting of X-ray absorption results**. The EXAFS curves of the Ru *K*-edge experimental data (denoted as Exp) and fitting results (denoted as Fit) of Ru/$TiO_x$ before and after OER in $k^3$-weighted *k*-space (**a**,**b**) and *R* space (**c**,**d**).

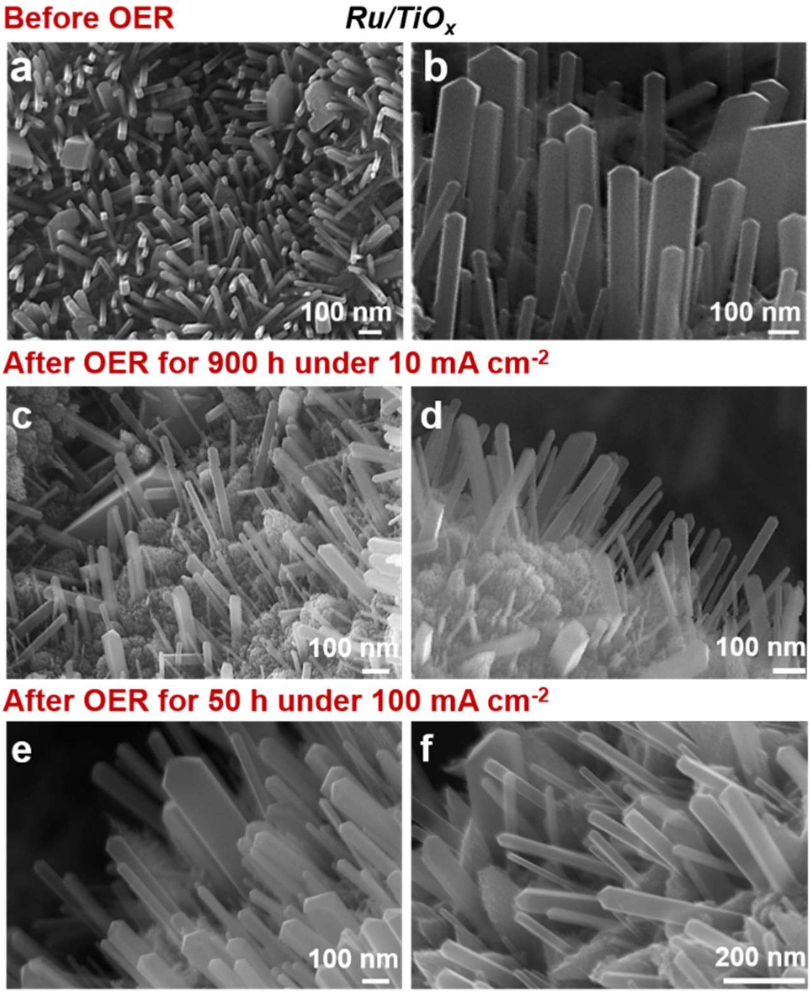


**Supplementary Figure 31. SEM images of Ru/TiO$_x$ before and after OER stability tests.** SEM images of Ru/TiO$_x$ (**a**,**b**) and Ru/TiO$_x$ after the chronoamperometric test under 10 mA cm$^{-2}$ for 900 h (**c**,**d**) and 100 mA cm$^{-2}$ for 50 h (**e**,**f**).

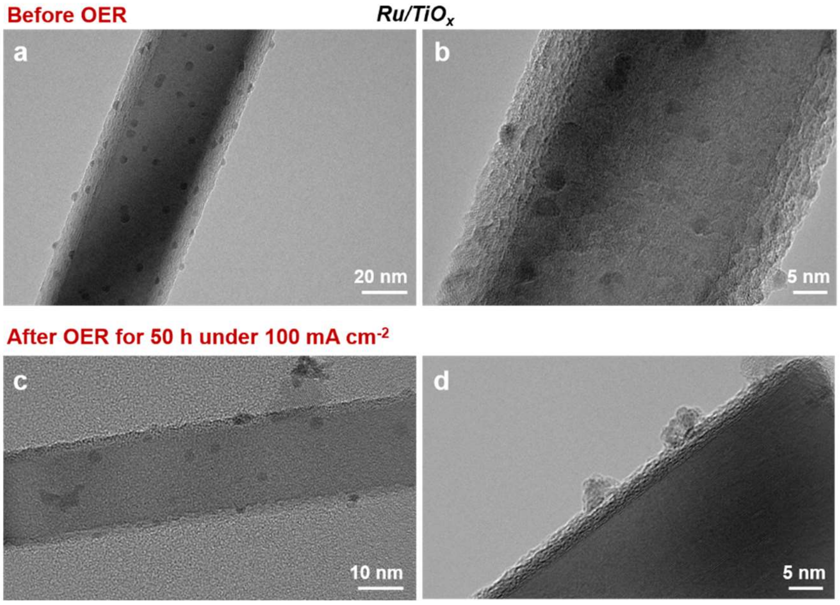


**Supplementary Figure 32. TEM images of $Ru/TiO_x$ before and after OER stability tests.** TEM images of $Ru/TiO_x$ (**a**,**b**) and $Ru/TiO_x$ after the chronoamperometric test under 100 mA $cm^{-2}$ for 50 h (**c**,**d**).

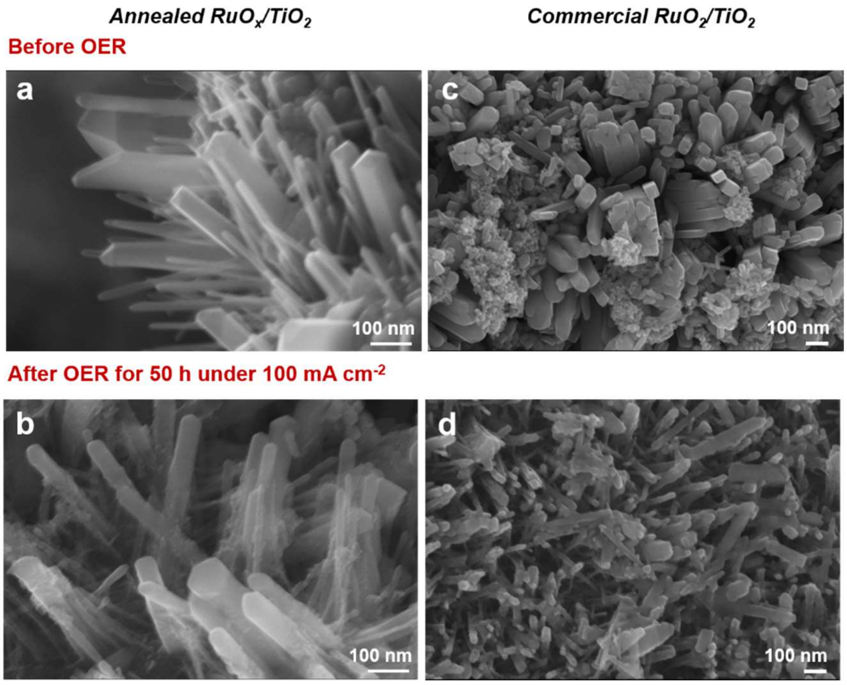


**Supplementary Figure 33. SEM images of control samples before and after OER stability tests.** **a**,**b**, SEM images of annealed $RuO_x/TiO_2$ before and after OER under 100 mA $cm^{-2}$ for 50 h. **c**,**d**, SEM images of commercial $RuO_x/TiO_2$ before and after the chronoamperometric test under 100 mA $cm^{-2}$ for 50 h.

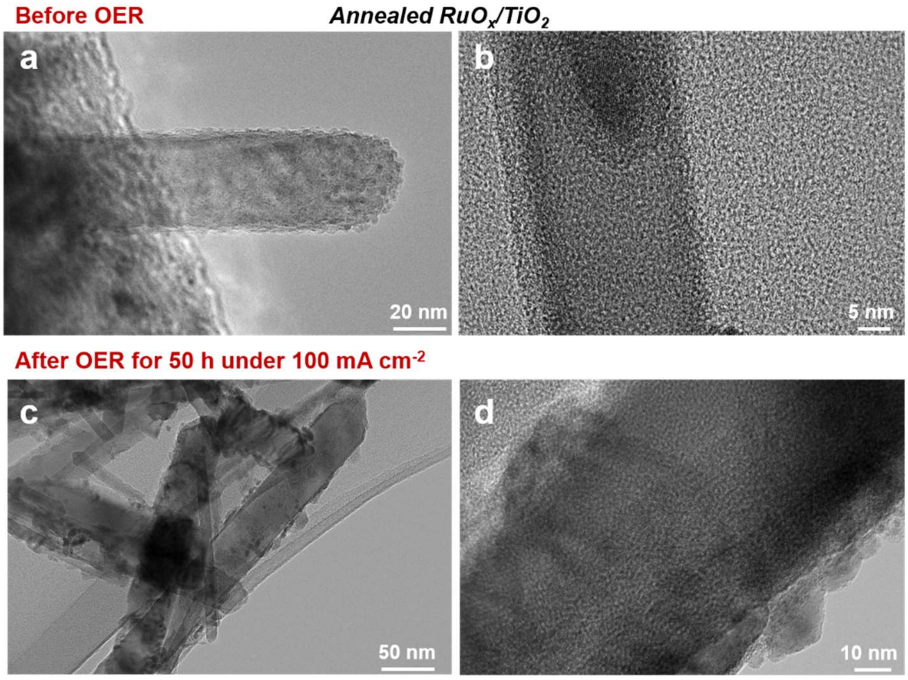


**Supplementary Figure 34. TEM images of control samples before and after OER stability tests.** TEM images of Ru/$TiO_x$ (**a**,**b**) and Ru/$TiO_x$ after the chronoamperometric test under 100 mA cm$^{-2}$ for 50 h (**c**,**d**).

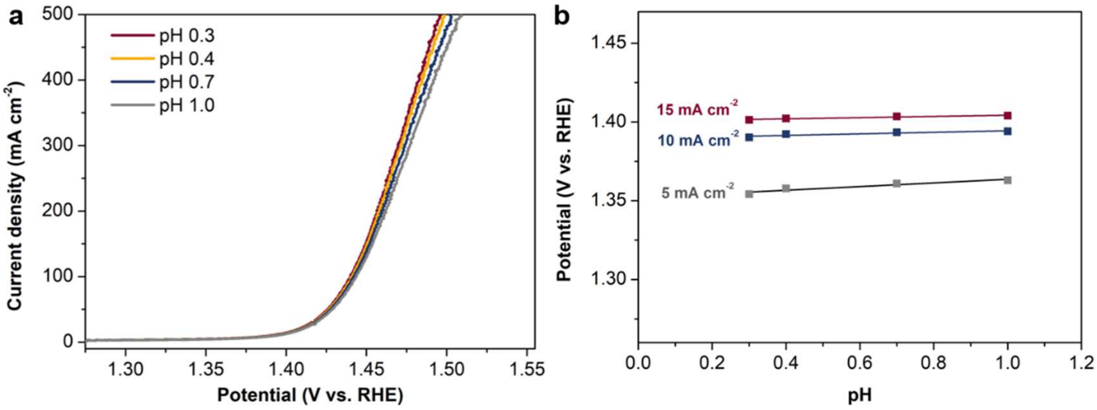


**Supplementary Figure 35. pH-dependence experiment. a**, OER activity of Ru/$TiO_x$ with varying pH. **b**, pH dependence on the OER potential at different current densities for Ru/$TiO_x$.

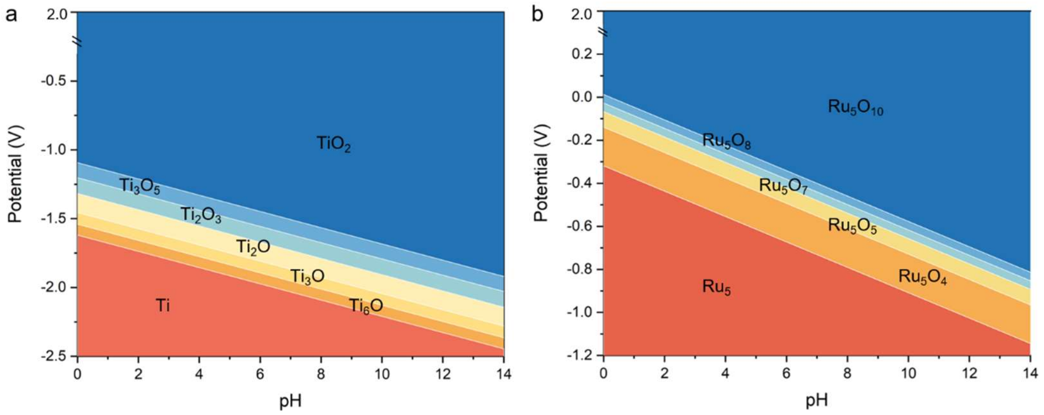


**Supplementary Figure 36.** Calculated Pourbaix diagrams of Ti (**a**) and Ru (**b**) systems.

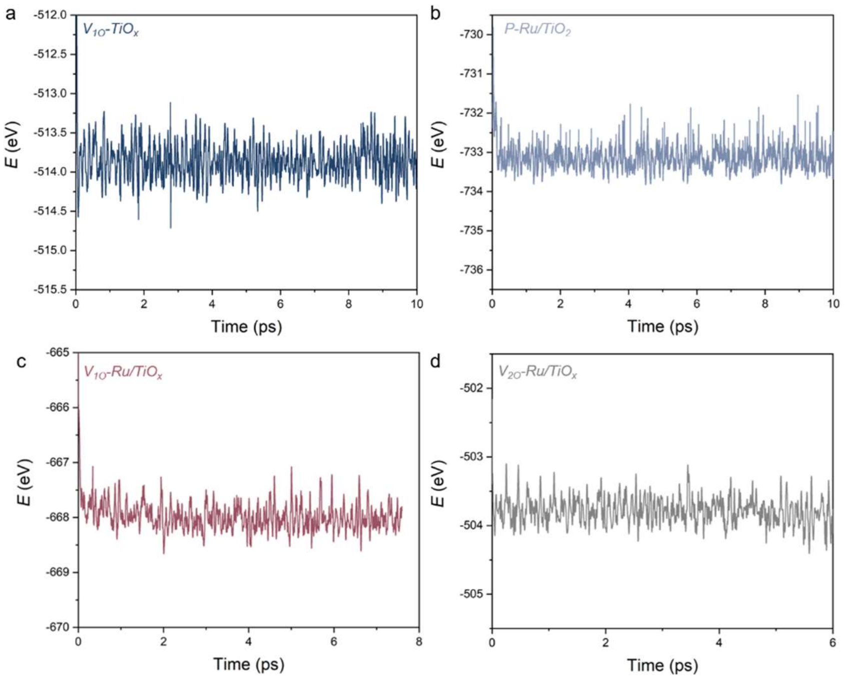


**Supplementary Figure 37.** The total energy of $V_{1O}$-$TiO_2$ (**a**), P-Ru/$TiO_2$ (**b**), $V_{1O}$-Ru/$TiO_x$ (**c**) and $V_{2O}$-Ru/$TiO_x$ (**d**) as a function of molecular dynamic (MD) time at a temperature of 300 K. ($V_{1O}$ and $V_{2O}$ denotes 1 and 2 oxygen vacancies, respectively; P denotes perfect structure without oxygen vacancy)

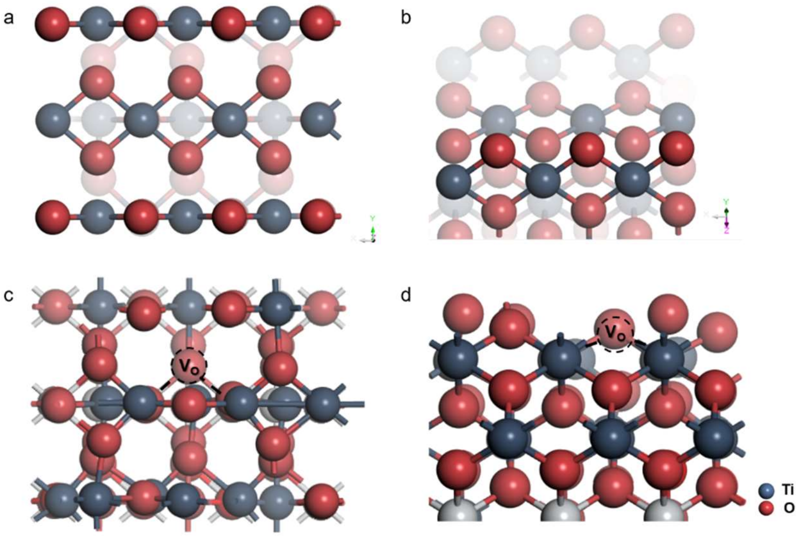


**Supplementary Figure 38. Theoretical calculation models of P-$TiO_2$ and V-$TiO_x$**. Top view and side view of P-$TiO_2$ (**a,b**) and $V_{1O}$-$TiO_x$ (**c,d**), respectively. (The blue and red balls represent Ti and O atoms, respectively).

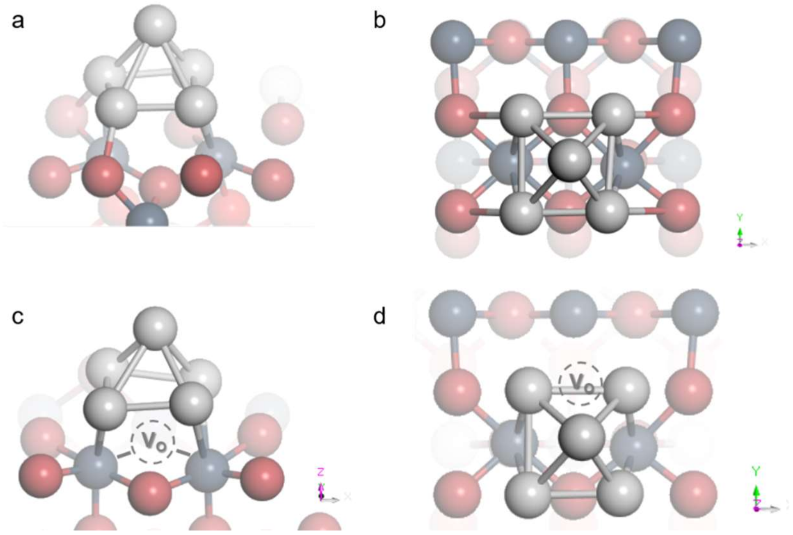


**Supplementary Figure 39. Theoretical calculation models of P-Ru/$TiO_2$ and $V_{1O}$-Ru/$TiO_x$**. Top view and side view of P-Ru/$TiO_2$ (**a,b**) and $V_{1O}$-$TiO_x$ (**c,d**), respectively. (The gray, blue and red balls represent Ru, Ti and O atoms, respectively).

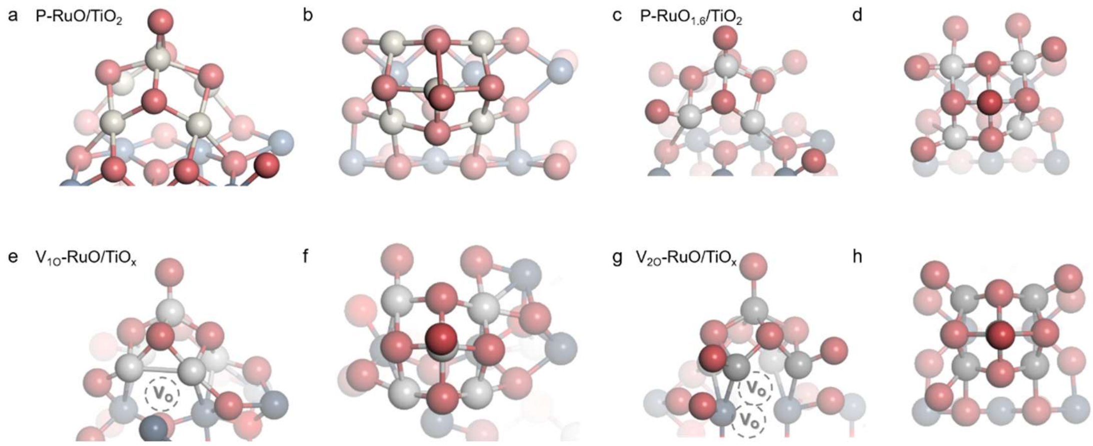


**Supplementary Figure 40. Theoretical calculation models of $RuO_x/TiO_x$.** Top view and side view of P-RuO/$TiO_2$ (**a,b**), P-$RuO_{1.6}/TiO_2$ (**c,d**), $V_{1O}$-RuO/$TiO_x$ (**e,f**) and $V_{2O}$-RuO/$TiO_x$ (**g,h**), respectively. (The gray, blue and red balls represent Ru, Ti and O atoms, respectively).

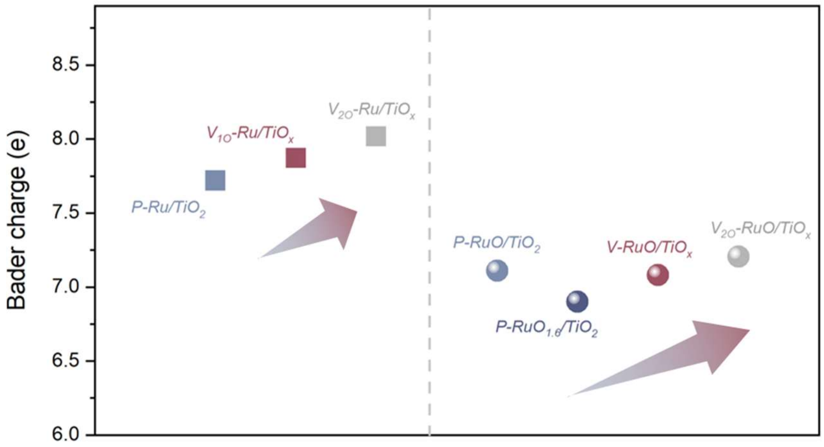


**Supplementary Figure 41.** Bader charge of interfacial Ru atom for different structures.

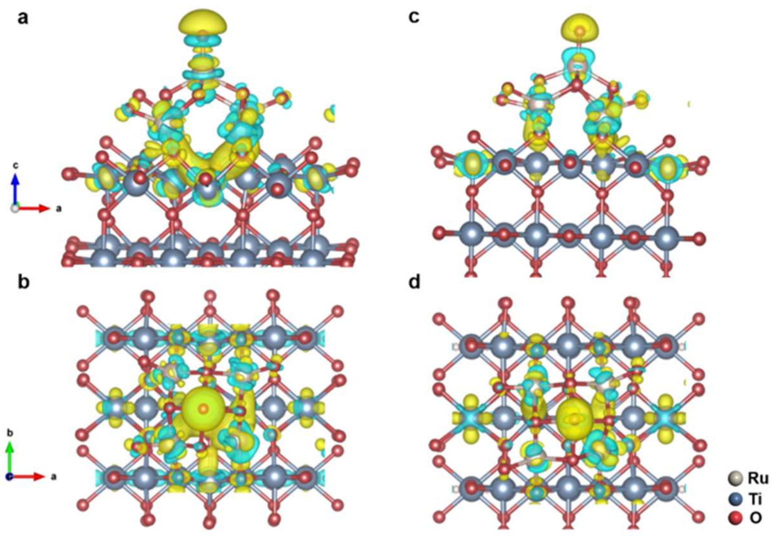


**Supplementary Figure 42.** Side view and top view of the differential charge density of $V_{1O}$-RuO/$TiO_x$ (**a,b**) and P-$RuO_{1.6}$/$TiO_2$ (**c,d**). Electron accumulation and depletion are shown in cyan and yellow, respectively. (isovalue is 0.01|e|/$Bohr^3$).

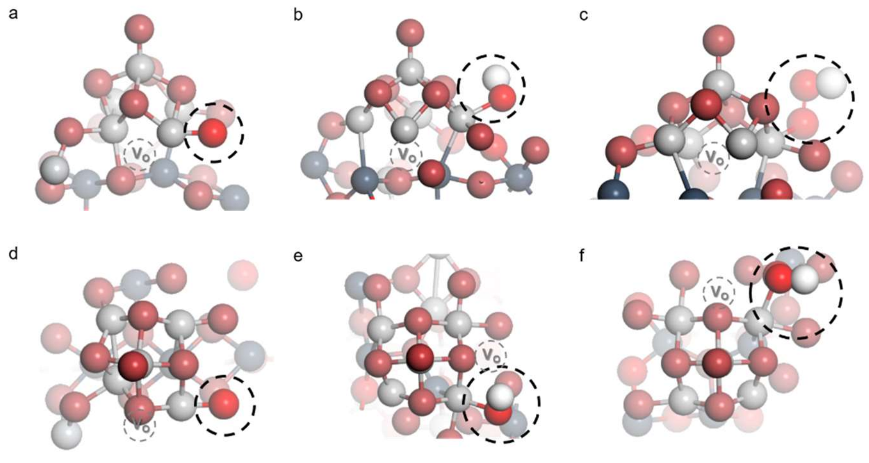


**Supplementary Figure 43.** Atomic structures of $V_{1O}$-RuO/$TiO_x$ with adsorbed OER intermediates. Side view (**a-c**) and top view (**d-f**) of $V_{1O}$-RuO/$TiO_x$ with adsorbed intermediate *O (**a,d**), *OH (**b,e**), and *OOH (**c,f**) on the interfacial Ru site.

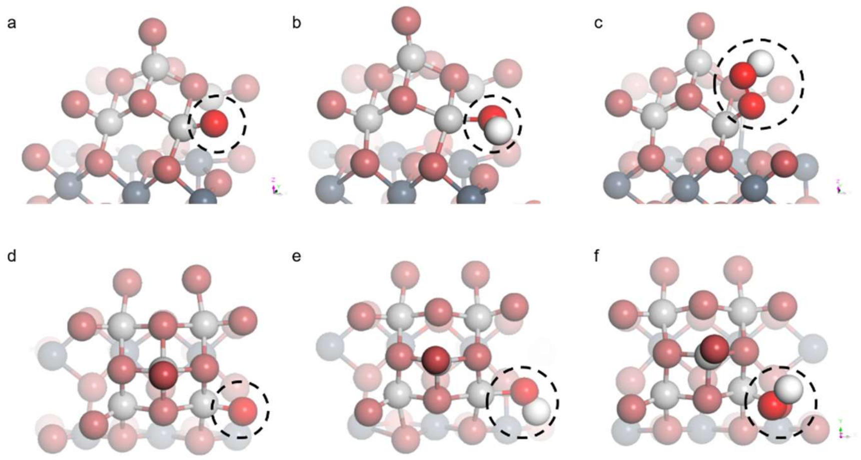


**Supplementary Figure 44.** Atomic structures of P-$RuO_{1.6}$/$TiO_2$ with adsorbed OER intermediates. Side view (**a-c**) and top view (**d-f**) of P-$RuO_{1.6}$/$TiO_2$ with adsorbed intermediate *O (**a,d**), *OH (**b,e**), and *OOH (**c,f**) on the interfacial Ru site.

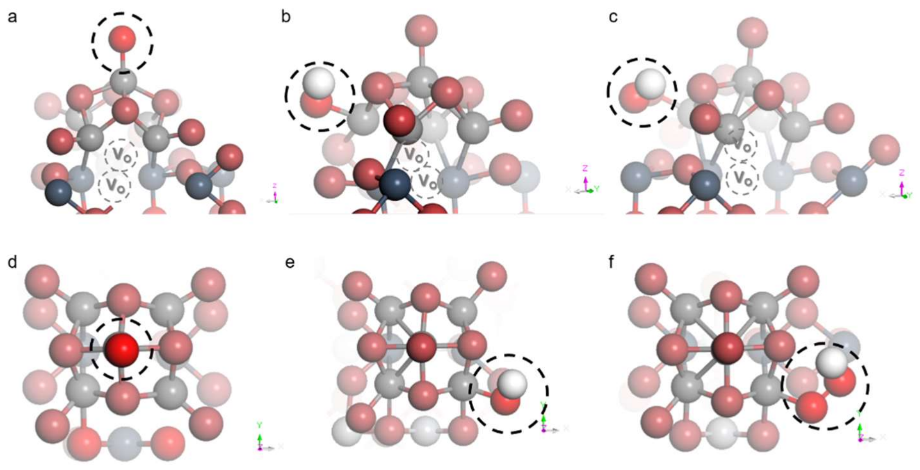


**Supplementary Figure 45**. Atomic structures of $V_{2O}$-RuO/$TiO_x$ with adsorbed OER intermediates. Side view (**a-c**) and top view (**d-f**) of $V_{2O}$-RuO/$TiO_x$ with adsorbed intermediate *O (**a**,**d**), *OH (**b**,**e**), and *OOH (**c**,**f**) on the interfacial Ru site.

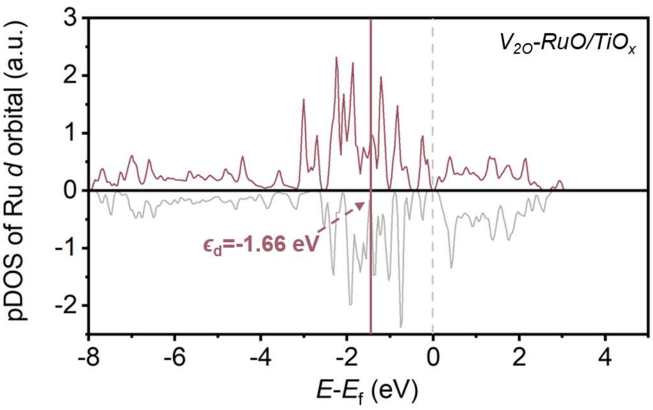


**Supplementary Figure 46.** Projected density of states (PDOS) and band center of Ru d-state for $V_{2O}$-RuO/$TiO_x$.

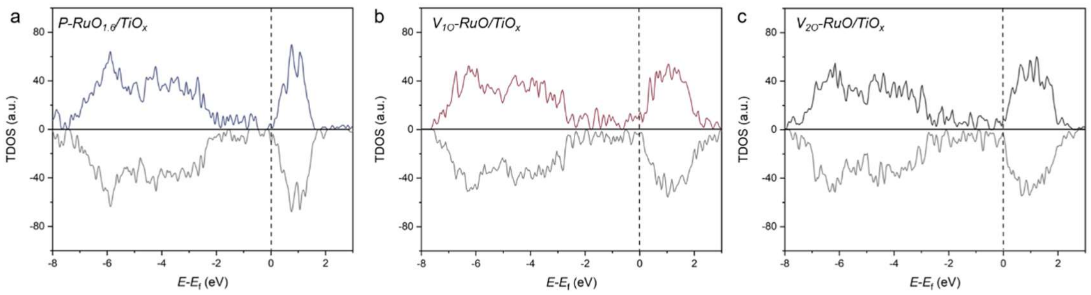


**Supplementary Figure 47.** Total density of states (TDOS) for P-$RuO_{1.6}$/$TiO_2$ (**a**), $V_{1O}$-RuO/$TiO_x$ (**b**) and $V_{2O}$-RuO/$TiO_x$ (**c**).

**Supplementary Table 1.** Summary of electrocatalytic OER performance of the Ru/$TiO_x$ catalysts and state-of-the-art electrocatalysts in acidic media.

| Samples | Electrolyte | $\eta_{10}$ | Stability | Reference |
|---|---|---|---|---|
| Ru/$TiO_x$ | 0.5 M $H_2SO_4$ | 174 | 900h @ 10 mA $cm^{-2}$; 50h @ 100 mA$cm^{-2}$ | This work |
| $RuO_2/TiO_2$ | 0.5 M $H_2SO_4$ | 208 | <1h @ 100 mA $cm^{-2}$ | This work |
| $IrO_2/TiO_2$ | 0.5 M $H_2SO_4$ | 236 | <10h @ 100 mA $cm^{-2}$ | This work |
| Ni-$RuO_2$ | 0.1 M $HClO_4$ | 214 | 200h @ 10 mA $cm^{-2}$ | [1]*Nat. Mater.* **22**, 100 (2023) |
| Ru@V-$RuO_2$/C | 0.5 M $H_2SO_4$ | 176 | 25h @ 10 mA $cm^{-2}$ | [2]*Adv. Mater.* **35**, 2206351 (2023) |
| C-$RuO_2$-RuSe | 0.5 M $H_2SO_4$ | 212 | 50h @ 20 mA $cm^{-2}$ 50h @ 50 mA $cm^{-2}$ | [3]*Chem* **8**, 1-15 (2022) |
| Ru/Co-N-C | 0.5 M $H_2SO_4$ | 232 | 20h @ 10 mA $cm^{-2}$ | [4]*Adv. Mater.* **34***,* 2110103 (2022) |
| PtCo–$RuO_2$/C | 0.1 M $HClO_4$ | 212.6 | 20h @ 10 mA $cm^{-2}$ | [5]*Energy Environ. Sci.* **15**, 1119 (2022) |
| $Ta_{0.1}Tm_{0.1}Ir_{0.8}O_{2-\delta}$ | 0.5 M $H_2SO_4$ | 226 | 500h @ 10 mA $cm^{-2}$ | [6]*Nat. Nanotech.* **16**, 1371 (2021) |
| 3R-$IrO_2$ | 0.1 M $HClO_4$ | 188 | 511h @ 10 mA $cm^{-2}$ | [7]*Joule* **5**, 3221 (2021) |
| Ir-$MnO_2$ | 0.5 M $H_2SO_4$ | 218 | 650h @ 10 mA $cm^{-2}$ | [8]*Joule* **5**, 2164 (2021) |
| E-Ru/Fe ONAs/C | 0.5 M $H_2SO_4$ | 238 | 9h @ 5 mA $cm^{-2}$ | [9]*Nano Energy* **84**, 105909 (2021) |
| $IrO_x$/9R-$BaIrO_3$ | 0.5 M $H_2SO_4$ | 230 | 48h @10 mA $cm^{-2}$ | [10]*J. Am. Chem. Soc.* **143**, 18001 (2021) |
| S-$RuFeO_x$ | 0.1 M $HClO_4$ | 187 | 50h @1 mA $cm^{-2}$ | [11]*Adv. Funct. Mater.* **31**, 2101405 (2021) |
| Ru/$RuS_2$ | 0.5 M $H_2SO_4$ | 201 | 24h @ 10 mA $cm^{-2}$ | [12]*Angew. Chem., Int. Ed.* **133**, 12436 (2021) |
| $RuNi_2$@G | 0.5 M $H_2SO_4$ | 227 | 24h @ 10 mA $cm^{-2}$ | [13]*Adv. Mater.* **32**, 1908126 (2020) |
| Ir−$NiCo_2O_4$ | 0.5 M $H_2SO_4$ | 240 | 70h @ 10 mA $cm^{-2}$ | [14]*J. Am. Chem. Soc.* **142**, 18378 (2020) |
| $Ru_1$–$Pt_3Cu$ | 0.1 M $HClO_4$ | 280 | 28h @ 10 mA $cm^{-2}$ | [15]*Nat. Catal.* **2**, 304-313 (2019) |
| Ru@$IrO_x$ | 0.05 M $H_2SO_4$ | 282 | 24h @ 1.55 V vs.RHE | [16]*Chem* **5**, 445-459 (2019) |
| $Cr_{0.6}Ru_{0.4}O_2$ | 0.5 M $H_2SO_4$ | 178 | 10h @ 10 mA $cm^{-2}$ | [17]*Nat. Commun*. **10**, 162 (2019) |
| RuCu nanosheets | 0.5 M $H_2SO_4$ | 236 | 13.5h @ 5 mA $cm^{-2}$ | [18]*Angew. Chem. Int. Ed.* **58**, 13983 (2019) |
| IrCo@$IrO_{x-n}$ NDs | 0.5 M $H_2SO_4$ | 247 | 10h @10 mA $cm^{-2}$ | [19]*Adv. Mater.* **31**, 1903616 (2019) |
| Ir-based nanocages | 0.5 M $H_2SO_4$ | 226 | 15h @ 10 mA $cm^{-2}$ | [20]*Angew. Chem. Int. Ed.* **58**, 7244 (2019) |

**Supplementary Table 2**. The mass loading (mg $cm^{-2}$) and weight percent ($_{wt}$%) of noble metal in different samples (by ICP-MS measurement and EDS) and atomic percent ($_{at}$%) by XPS measurement.

| Noble metal in sample | Mass loading (mg $cm^{-2}$) | Weight % ($_{wt}$%) (ICP) | Weight % ($_{wt}$%) (EDS) | Atomic % ($_{at}$%) (XPS) |
|---|---|---|---|---|
| Ru/$TiO_x$ | 0.0715 | 0.075 | 7.7% | 9.3% |
| Annealed $RuO_x$/$TiO_2$ | 0.0867 | 0.083 | 8.2% | 9.2% |
| Com. $RuO_2$/$TiO_2$ | 0.0992 | 0.092 | - | 10.1% |
| Com. $IrO_2$/$TiO_2$ | 0.1135 | 0.107 | 8.8% | 7.5% |
| Ru/$TiO_x$ after OER | 0.0695 | 0.073 | 6.4% | 7.2% |
| Annealed $RuO_x$/$TiO_2$ after OER | 0.0367 | 0.035 | 3.3% | 4.3% |
| Com. $RuO_2$/$TiO_2$ after OER | 0.0201 | 0.019 | - | 2.4% |

*Note: Since the catalysts are binder-free electrodes, the catalysts are dissolved together with the substrates during the ICP test, while EDS and XPS only detect the surface content, so the $_{wt}$% obtained through ICP is less than that obtained through EDS and XPS, but the trend is consistent.

**Supplementary Table 3**. OER mass activity comparison between the as-synthesized $Ru/TiO_x$ catalyst and other reported noble metal-based electrocatalysts in acidic media.

| Catalysts | Electrolyte | Potential (V vs. RHE) | Mass activity (A $g_{noble\ metal}^{-1}$) | Reference |
|---|---|---|---|---|
| $Ru/TiO_x$ | 0.5 M $H_2SO_4$ | 1.45 | 2128.2 | This work |
| | | 1.5 | 5876.4 | |
| Com. $RuO_2/TiO_2$ | 0.5 M $H_2SO_4$ | 1.45 | 462.0 | This work |
| | | 1.5 | 1062.9 | |
| Com. $IrO_2/TiO_2$ | 0.5 M $H_2SO_4$ | 1.45 | 46.0 | This work |
| | | 1.5 | 191.7 | |
| Dealloyed nanoporous IrNi | 0.5 M $H_2SO_4$ | 1.5 | 52.5 | [21]*Energy Environ. Sci.* **15**, 3449-3461 (2022) |
| $Ir-MnO_2$ | 0.5 M $H_2SO_4$ | 1.53 | 766.0 | [8]*Joule* **8**, 1-8 (2021) |
| $Ru/MnO_2$ | 0.1 M $HClO_4$ | 1.40 | 1264.0 | [22]*Nat. Catal.* **4**, 1012-1023 (2021) |
| RuIr nanosized-coral | 0.05 M $H_2SO_4$ | 1.45 | 796.0 | [23]*Nat Commun.* **12**, 1145 (2021) |
| RuIr@carbon support | 0.5 M $H_2SO_4$ | 1.53 | 2041.0 | [24]*ACS Catal.* **11**, 3402-3413 (2021) |
| IrCuNi deeply concave nanocubes/C | 0.1 M $HClO_4$ | 1.53 | 6600.0 | [25]*Nano Lett.* **21**, 2809-2816 (2021) |
| Ru@Ir-O | 0.5 M $H_2SO_4$ | 1.55 | 1169.0 | [26]*Small* **18**, 2108031 (2022) |
| $S-RuFeO_x$ | 0.1 M $HClO_4$ | 1.42 | 1180.0 | [11]*Adv. Funct. Mater.* **31**, 2101405 (2021) |
| $RuO_2/(Co,Mn)_3O_4/CC$ | 0.5 M $H_2SO_4$ | 1.53 | 366.5 | [27]*Appl. Catal., B* **31**, 2101405 (2021) |
| Ir-Ta NPs | 0.1 M $HClO_4$ | 1.55 | 650±150 | [28]*Nat. Energy* **7**, 55-64 (2022) |
| Atomically dispersed hetero-nitrogen Ir | 0.5 M $H_2SO_4$ | 1.45 | 2860.0 | [29]*Nat. Commun.,* **12**, 6118 (2021) |
| $RuO_2$-nanosheets/carbon fiber | 0.5 M $H_2SO_4$ | 1.53 | 115.5 | [30]*Nano Energy* **88**, 106276 (2021) |
| $Ni-Ru@RuO_x$ | 0.5 M $H_2SO_4$ | 1.45 | 315.0 | [31]*Adv. Energy Mater.* **11**, 2003448 (2021) |
| PdCu/Ir/C | 0.1 M $HClO_4$ | 1.51 | 1190 | [32]*Angew* **60**, 8243-8250 (2021) |

**Supplementary Table 4.** TOF of Ru/$TiO_x$ with previously reported OER catalysts in acid.

| Catalysts | Electrolyte | Overpotential (mV) | TOF ($s^{-1}$) | Reference |
|---|---|---|---|---|
| Ru/$TiO_x$ | 0.5 M $H_2SO_4$ | 270 | 1.707 | This work |
| | | 300 | 1.960 | |
| Annealed $RuO_x$/$TiO_2$ | 0.5 M $H_2SO_4$ | 300 | 0.820 | This work |
| Com. $RuO_2$ | 0.5 M $H_2SO_4$ | 300 | 0.322 | This work |
| $SnRuO_x$ | 0.5 M $H_2SO_4$ | 250 | 0.63 | [33]*Nat. Commun*. **14**, 843 (2023) |
| Rh-$RuO_2$/Graphene | 0.5 M $H_2SO_4$ | 300 | 1.74 | [34]*Nat. Commun*. **14**, 1412 (2023) |
| high-loading Ir single atoms with *d*-band holes | 0.1 M $HClO_4$ | 216 | 0.599 | [53]*Angew. Chem., Int. Ed.* **135**, 202308082 (2023) |
| $Ru_5W_1O_x$ | 0.5 M $H_2SO_4$ | 300 | 0.163 | [36]*Nat. Commun*. **13**, 4871 (2022) |
| Cr-$SrIrO_3$ | 0.1 M $HClO_4$ | 300 | 0.208 | [37]*Nano Energy* **102**, 107680 (2022) |
| Ru/$MnO_2$ | 0.1 M $HClO_4$ | 165 | 0.331 | [38]*Nat. Catal.* **4**, 1012-1023 (2021) |
| $Ru_1Ir_1O_x$ | 0.5 M $H_2SO_4$ | 300 | 0.47 | [39]*Adv. Energy Mater*. **11**, 2102883 (2021) |

**Supplementary Table 5.** TOF of catalysts using different normalization methods.

| Catalysts | Overpotential (mV) | Bulk TOF ($s^{-1}$) | ECSA TOF ($s^{-1}$) |
|---|---|---|---|
| Ru/$TiO_x$ | 270 | 1.707 | 1.835 |
| | 300 | 1.960 | 2.192 |
| Annealed $RuO_x$/$TiO_2$ | 300 | 0.820 | 1.640 |
| Com. $RuO_2$ | 300 | 0.322 | 1.520 |

**Supplementary Table 6.** Comparison of the PEM electrolyzer performance with those previously reported.

| Anode catalysts | Cell voltage (V) | Stability | Reference |
|---|---|---|---|
| $Ru/TiO_x$ | 1.71 V @ 1 A $cm^{-2}$ | 0.5 A $cm^{-2}$ for 200 h | This work |
| $RuO_2$ | 1.94 V @ 1 A $cm^{-2}$ | 0.5 A $cm^{-2}$ for < 50 h | This work |
| $Nb_{0.1}Ru_{0.9}O_2$ | 1.69 V @ 1 A $cm^{-2}$ | 0.3 A $cm^{-2}$ for 100 h | [40]*Joule* **7**, 558-573 (2023) |
| $Y_2MnRuO_7$ | 1.51 V @ 0.2 A $cm^{-2}$ | 0.2 A $cm^{-2}$ for 24 h | [41]*Nat. Commun*. **14**, 2010 (2023) |
| $Nd_{0.1}RuOx$ | 1.595 V @ 0.05 A $cm^{-2}$ | 0.05 A $cm^{-2}$ for 50 h | [42]*Adv. Funct. Mater.* **33**, 2213304 (2023) |
| $IrO_x/Zr_2ON_2$ | 1.927 V at 2.0 A $cm^{-2}$ | 1.0 A $cm^{-2}$ for 50 h | [43]*Adv. Funct. Mater.* **33**, 2301557 (2023) |
| $RuO_2$/Defect-$TiO_2$ | 1.74 V @ 1.5 A $cm^{-2}$ | 1.0 A $cm^{-2}$ for 6 h | [44]*ACS Catal*. **12**, 9437-9445 (2022) |
| Strained-$RuO_2$/ATO | 1.51 V @ 1 A $cm^{-2}$ | 0.5 A $cm^{-2}$ for 40 h | [45]*Adv. Sci*. **9**, 2201654 (2022) |
| $W_{0.2}Er_{0.1}Ru_{0.7}O_{2-\delta}$ | - | 0.1 A $cm^{-2}$ for 120 h | [46]*Nat. Commun*. **11**, 5368 (2020) |

**Supplementary Table 7.** Concentrations of the major constituents in natural seawater.

| Species | Concentration (ppm) |
|---|---|
| $Cl^-$ | 19350 |
| $Na^+$ | 9685 |
| $SO_4^{2-}$ | 2410 |
| $Mg^{2+}$ | 870 |
| $Ca^{2+}$ | 344 |

**Supplementary Table 8.** High resolution Ru 3*d* XPS peak fitting parameters of different samples before and after OER.

| Sample | Core level | Peak position (eV) | Peak area | FWHM (eV) [a)] |
|---|---|---|---|---|
| Ru/$TiO_x$ | Ru $3d_{5/2}$ | 280.6 | 34594.87 | 0.68 |
| | | 281.16 | 25185.03 | 1.57 |
| | Ru $3d_{3/2}$ | 284.7 | 23063.65 | 0.93 |
| | | 285.25 | 16790.02 | 1.81 |
| | C 1*s* | 284.8 | 27369.31 | 1.81 |
| Annealed $RuO_x/TiO_2$ | Ru $3d_{5/2}$ | 280.62 | 67103.88 | 0.98 |
| | | 282.33 | 55862.16 | 1.78 |
| | Ru $3d_{3/2}$ | 284.72 | 44735.92 | 0.98 |
| | | 286.43 | 37241.44 | 1.82 |
| | C 1*s* | 284.8 | 34397.01 | 1.77 |
| Com. $RuO_2/TiO_2$ | Ru $3d_{5/2}$ | 280.65 | 14552.20 | 0.92 |
| | | 282.43 | 15022.85 | 1.82 |
| | Ru $3d_{3/2}$ | 284.75 | 9701.47 | 1.02 |
| | | 286.53 | 10015.23 | 1.85 |
| | C 1*s* | 284.8 | 6377.99 | 1.91 |
| Ru/$TiO_x$ after OER | Ru $3d_{5/2}$ | 280.61 | 21619.56 | 1.41 |
| | | 282.33 | 15987.43 | 1.91 |
| | Ru $3d_{3/2}$ | 284.72 | 14413.04 | 1.58 |
| | | 286.44 | 10658.29 | 1.9 |
| | C 1*s* | 284.8 | 9130.65 | 1.98 |
| Annealed $RuO_x/TiO_2$ after OER | Ru $3d_{5/2}$ | 281.16 | 14047.01 | 0.95 |
| | | 282.38 | 15072.05 | 1.81 |
| | Ru $3d_{3/2}$ | 285.26 | 9364.67 | 1.05 |
| | | 286.48 | 10048.03 | 1.89 |
| | C 1*s* | 284.8 | 28387.34 | 1.5 |
| Com. $RuO_2/TiO_2$ after OER | Ru $3d_{5/2}$ | 281.55 | 12506.05 | 1.23 |
| | | 282.71 | 16144.38 | 1.47 |
| | Ru $3d_{3/2}$ | 285.65 | 8337.36 | 1.26 |
| | | 286.8 | 10762.92 | 1.65 |
| | C 1*s* | 284.8 | 56728.58 | 1.27 |

[a)] FWHM: full-width at the half of the maximum.

**Supplementary Table 9.** High resolution Ru 3*d*, Ti 2*p* and O 1*s* XPS peak fitting parameters of $Ru/TiO_x$ at applied potential during 1.0-1.7 V vs. RHE.

| Sample | Core level | Peak position (eV) | Peak area | FWHM (eV) [a)] |
|---|---|---|---|---|
| $Ru/TiO_x$-1.0 | Ru $3d_{5/2}$ | 280.12 | 2593.16 | 1.07 |
| | | 280.77 | 1836.78 | 1.17 |
| | Ru $3d_{3/2}$ | 284.22 | 1728.77 | 1.11 |
| | | 284.87 | 1224.52 | 1.19 |
| | Ti $2p_{3/2}$ | 457.90 | 7399.48 | 1.20 |
| | | 458.51 | 23726.30 | 1.14 |
| | Ti $2p_{1/2}$ | 463.90 | 3699.74 | 1.21 |
| | | 464.51 | 11863.15 | 1.54 |
| | Ru $3p_{3/2}$ | 460.60 | 1470.22 | 1.90 |
| | | 462.96 | 2533.76 | 1.48 |
| | O 1*s* | 529.52 | 19079.02 | 1.88 |
| | | 530.45 | 9727.79 | 1.45 |
| | | 531.47 | 8456.91 | 1.79 |
| $Ru/TiO_x$-1.2 | Ru $3d_{5/2}$ | 280.13 | 2734.80 | 1.05 |
| | | 280.81 | 1969.02 | 1.20 |
| | Ru $3d_{3/2}$ | 284.23 | 1823.20 | 1.07 |
| | | 284.91 | 1312.68 | 1.21 |
| | Ti $2p_{3/2}$ | 457.90 | 7001.99 | 1.23 |
| | | 458.58 | 22629.02 | 1.09 |
| | Ti $2p_{1/2}$ | 463.90 | 3500.99 | 1.23 |
| | | 464.50 | 11314.51 | 1.49 |
| | Ru $3p_{3/2}$ | 460.60 | 1591.89 | 1.89 |
| | | 462.95 | 2534.37 | 1.68 |
| | O 1*s* | 529.55 | 19282.80 | 1.86 |
| | | 530.40 | 9066.99 | 1.51 |
| | | 531.43 | 8160.46 | 1.89 |
| $Ru/TiO_x$-1.4 | Ru $3d_{5/2}$ | 280.15 | 2678.28 | 1.07 |
| | | 280.83 | 1945.85 | 1.44 |
| | Ru $3d_{3/2}$ | 284.25 | 1785.52 | 1.08 |
| | | 284.93 | 1297.23 | 1.45 |
| | Ti $2p_{3/2}$ | 457.90 | 3294.47 | 1.16 |
| | | 458.54 | 15788.83 | 1.14 |
| | Ti $2p_{1/2}$ | 463.90 | 1647.24 | 1.16 |

| | | | | |
|---|---|---|---|---|
| | | 464.54 | 7894.42 | 1.54 |
| | Ru 3$p_{3/2}$ | 460.90 | 1172.22 | 1.98 |
| | | 463.08 | 1976.27 | 1.67 |
| | O 1$s$ | 529.60 | 20864.25 | 1.72 |
| | | 530.48 | 9731.28 | 1.41 |
| | | 531.49 | 8407.48 | 1.98 |
| Ru/$TiO_x$-1.6 | Ru 3$d_{5/2}$ | 280.17 | 2435.83 | 1.03 |
| | | 280.95 | 1688.01 | 1.37 |
| | Ru 3$d_{3/2}$ | 284.27 | 1557.22 | 1.05 |
| | | 285.05 | 1218.68 | 1.37 |
| | Ti 2$p_{3/2}$ | 457.90 | 5249.07 | 0.98 |
| | | 458.60 | 29718.37 | 1.10 |
| | Ti 2$p_{1/2}$ | 463.90 | 2624.53 | 0.98 |
| | | 464.60 | 14859.18 | 1.50 |
| | Ru 3$p_{3/2}$ | 460.40 | 2316.29 | 1.92 |
| | | 463.15 | 3039.33 | 1.52 |
| | O 1$s$ | 529.67 | 21524.80 | 1.63 |
| | | 530.48 | 9988.22 | 1.38 |
| | | 531.52 | 7457.96 | 1.89 |
| Ru/$TiO_x$-1.7 | Ru 3$d_{5/2}$ | 280.21 | 2506.04 | 1.04 |
| | | 281.00 | 1826.04 | 1.35 |
| | Ru 3$d_{3/2}$ | 284.31 | 1670.69 | 1.04 |
| | | 285.10 | 1217.36 | 1.35 |
| | Ti 2$p_{3/2}$ | 457.90 | 5156.78 | 1.12 |
| | | 458.63 | 29558.12 | 1.07 |
| | Ti 2$p_{1/2}$ | 463.90 | 2578.39 | 1.12 |
| | | 464.63 | 14779.06 | 1.47 |
| | Ru 3$p_{3/2}$ | 460.60 | 2411.36 | 1.98 |
| | | 463.27 | 3225.89 | 1.89 |
| | O 1$s$ | 526.69 | 23711.47 | 1.56 |
| | | 530.46 | 11822.26 | 1.27 |
| | | 531.50 | 8267.88 | 2.03 |

[a)]FWHM: full-width at the half of the maximum.

**Supplementary Table 10**. Summary of Ru *K*-edge adsorption energy ($E_0$) and valence states for Ru foil, $RuO_2$, Ru/$TiO_x$ before and after OER.

| Sample | Ru *K*-edge Energy (eV) | Ru Valence State |
|---|---|---|
| Ru foil | 22115.01 | 0 |
| Ru/$TiO_x$ | 22116.21 | +0.34 |
| Ru/$TiO_x$ after OER | 22117.36 | +0.72 |
| $RuO_2$ | 22127.98 | 4 |

**Supplementary Table 11**. Structural parameters obtained from the curve-fitting analysis of the Ru *K*-edge EXAFS spectra.

| Sample | Path | $N^{a)}$ | $R$ (Å)$^{b)}$ | $\sigma^2$ ($10^{-3}$Å$^2$)$^{c)}$ | $\Delta E_0$ (eV)$^{d)}$ | *R-factor*$^{e)}$ |
|---|---|---|---|---|---|---|
| Ru foil | Ru-Ru | 12 | 2.67 ±0.01 | 3.10 ±0.2 | -5.80±0.7 | 0.010 |
| $RuO_2$ | Ru-Ru | 2 | 3.14 ±0.01 | 0.80 ±0.4 | -3.60±0.8 | 0.011 |
| | Ru-O | 6 | 1.97 ±0.01 | 3.30 ±0.3 | -3.60 ±0.8 | |
| Ru/$TiO_x$ | Ru-Ru | 7.8 | 2.66±0.01 | 3.56 ±1.0 | -5.60±0.6 | 0.017 |
| | Ru-O | 1.8 | 2.05±0.02 | 2.46 ±0.8 | -6.10±1.0 | |
| Ru/$TiO_x$ after OER | Ru-Ru | 7.4 | 2.68±0.01 | 3.55 ±1.0 | -3.90±1.0 | 0.019 |
| | Ru-O | 2.0 | 2.02±0.02 | 4.50 ±0.8 | -5.70±0.8 | |

a) $N$: coordination number; b) $R$: bond distance; c) $\sigma^2$: Debye-Waller factor; d) $\Delta E_0$: inner potential correction; e) *R-factor*: goodness of fit.

**Supplementary Table 12**. Integrated COHP (ICOHP) value for adsorption *O of Ru-O in different models.

| Model | -ICOHP |
|---|---|
| P-$RuO_x$/$TiO_2$ | -7.61 |
| V-$RuO_x$/$TiO_x$ | -7.50 |

## Supplementary References


1. Wu, Z.-Y. et al. Non-iridium-based electrocatalyst for durable acidic oxygen evolution reaction in proton exchange membrane water electrolysis. *Nat. Mater*. **22**, 100-108 (2023).
2. Li, Y.-P. et al. Arming Ru with oxygen-vacancy-enriched $RuO_2$ sub-nanometer skin activates superior bifunctionality for pH-universal overall water splitting. *Adv. Mater.* **35**, 2206351 (2023).
3. Wang, J. et al. Exceptionally active and stable $RuO_2$ with interstitial carbon for water oxidation in acid. *Chem* **8**, 1673 (2022).
4. Rong, C.-L. et al. Electronic structure engineering of single-atom Ru Sites via Co-$N_4$ Sites for bifunctional pH-universal water splitting. *Adv. Mater.* **34**, 2110103 (2022).
5. Jin, H. et al. Safeguarding the $RuO_2$ phase against lattice oxygen oxidation during acidic water electrooxidation. *Energy Environ. Sci.* **15**, 1119-1130 (2022).
6. Wang, J. et al. Exceptionally active and stable $RuO_2$ with interstitial carbon for water oxidation in acid. *Chem* **8**, 1673 (2022).
7. Hao, S.-Y. et al. Torsion strained iridium oxide for efficient acidic water oxidation in proton exchange membrane electrolyzers. *Nat. Nanotech*. **16**, 1371-1377 (2021).
8. Fan, Z. L. et al. Extraordinary acidic oxygen evolution on new phase 3R-iridium oxide. *Joule* **5**, 3221-3234 (2021).
9. Shi, Z. P. et al. Confined Ir single sites with triggered lattice oxygen redox: toward boosted and sustained water oxidation catalysis. *Joule* **5**, 2164-2176 (2021).
10. Yao, Q., Huang B.-L., Xu, Y., Li, L.-G., Shao, Q. & Huang, X.-H. A chemical etching strategy to improve and stabilize $RuO_2$-based nanoassemblies for acidic oxygen evolution. *Nano Energy* **84**, 105909 (2021).
11. Li, N. et al. Identification of the active-layer structures for acidic oxygen evolution from 9R-$BaIrO_3$ electrocatalyst with enhanced iridium mass activity. *J. Am. Chem. Soc.* **143**, 18001-18009 (2021).
12. Xue, Y.-R. et al. Sulfate-functionalized $RuFeO_x$ as highly efficient oxygen evolution reaction electrocatalyst in acid. *Adv. Funct. Mater.* **31**, 2101405 (2021).
13. Zhu, J.-W. et al. Regulative electronic states around ruthenium/ruthenium disulphide

heterointerfaces for efficient water splitting in acidic media. *Angew. Chem., Int. Ed.* **133**, 12436-12442 (2021).

14. Cui, X.-J. et al. Robust interface Ru centers for high-performance acidic oxygen evolution. *Adv Mater*. **32**, 1908126 (2020).
15. Yin, J. et al. Iridium single atoms coupling with oxygen vacancies boosts oxygen evolution reaction in acid media. *J. Am. Chem. Soc.* **142**, 18378-18386 (2020).
16. Yao, Y.-C. et al. Engineering the electronic structure of single atom Ru sites via compressive strain boosts acidic water oxidation electrocatalysis. *Nat. Catal.* **2**, 304-313 (2019).
17. Shan, J.-Q. et al. Charge-redistribution-enhanced nanocrystalline Ru@$IrO_x$ electrocatalysts for oxygen evolution in acidic media. *Chem* **5**, 445-459 (2019).
18. Lin, Y. C. et al. Chromium-ruthenium oxide solid solution electrocatalyst for highly efficient oxygen evolution reaction in acidic media. *Nat. Commun*. **10**, 162 (2019).
19. Yao, Q., Huang, B.-L., Zhang, N., Sun, M.-Z., Shao, Q. & Huang X.-Q. Channel-rich RuCu nanosheets for pH-universal overall water splitting electrocatalysis. *Angew. Chem., Int. Ed.* **58**, 13983-13988 (2019).
20. Shan, J. Q., Ling, T., Davey, K., Zheng, Y. & Qiao, S.-Z. Transition-metal-doped RuIr bifunctional nanocrystals for overall water splitting in acidic environments. *Adv. Mater*. **31**, 1903616 (2019).
21. Zhu, J. et al. Iridium-based cubic nanocages with 1.1-nm-thick walls: a highly efficient and durable electrocatalyst for water oxidation in an acidic medium. *Angew. Chem., Int. Ed.* **58**, 7244 (2019).
22. Yeo, K.-R. et al. A highly active and stable 3D dandelion spore-structured self-supporting Ir-based electrocatalyst for proton exchange membrane water electrolysis fabricated using structural reconstruction. *Energy Environ. Sci.* **15**, 3449-3461 (2022).
23. Lin, C. et al. In-situ reconstructed Ru atom array on α-$MnO_2$ with enhanced performance for acidic water oxidation. *Nat. Catal.* **4**, 1012-1023 (2021).
24. Wu, D.-S. et al. Efficient overall water splitting in acid with anisotropic metal nanosheets. *Nat Commun.* **12**, 1145 (2021).
25. Xu, J.-Y. et al. Atomic-step enriched ruthenium-iridium nanocrystals anchored homogeneously on MOF-derived support for efficient and stable oxygen evolution in acidic

and neutral media. *ACS Catal.* **11**, 3402-3413 (2021).

26. Liu, D. et al. IrCuNi deeply concave nanocubes as highly active oxygen evolution reaction electrocatalyst in acid electrolyte. *Nano Lett.* **21**, 2809-2816 (2021).
27. Zhang, J.-H. et al. Core-shell nanostructured Ru@Ir-O electrocatalysts for superb oxygen evolution in acid. *Small* **18**, 2108031 (2022).
28. Niu, S.-Q. et al. Low Ru loading $RuO_2/(Co,Mn)_3O_4$ nanocomposite with modulated electronic structure for efficient oxygen evolution reaction in acid. *Appl. Catal. B* **297**, 120442 (2021).
29. Zheng, Y.-R. et al. Monitoring oxygen production on mass-selected iridium-tantalum oxide electrocatalysts. *Nat. Energy* **7**, 55-64 (2022).
30. Su, H. et al. In-situ spectroscopic observation of dynamic-coupling oxygen on atomically dispersed iridium electrocatalyst for acidic water oxidation. *Nat. Commun.,* **12**, 6118 (2021).
31. Huang, H.-W. et al. Structure engineering defective and mass transfer-enhanced $RuO_2$ nanosheets for proton exchange membrane water electrolyzer. *Nano Energy* **88**, 106276 (2021).
32. Harzandi, A. M. et al. Ruthenium core-shell engineering with nickel single atoms for selective oxygen evolution via nondestructive mechanism. *Adv. Energy Mater.* **11**, 2003448 (2021).
33. Li, M.-G. et al. Exclusive strain effect boosts overall water splitting in PdCu/Ir core/shell nanocrystals. *Angew. Chem., Int. Ed.* **60**, 8243-8250 (2021).
34. Shi, Z.-P. et al. Customized reaction route for ruthenium oxide towards stabilized water oxidation in high-performance PEM electrolyzers, *Nat. Commun*. **14**, 843 (2023).
35. Wang, Y. et al. Unraveling oxygen vacancy site mechanism of Rh-doped $RuO_2$ catalyst for long-lasting acidic water oxidation, *Nat. Commun*. **14**, 1412 (2023).
36. Zhang, X.-X. et al. Fast modulation of *d*-Band Holes Quantity in the Early Reaction Stages for Boosting Acidic Oxygen Evolution, *Angew. Chem., Int. Ed.* **135**, 202308082 (2023).
37. Wen, Y.-Z. et al. Introducing Brønsted acid sites to accelerate the bridging-oxygen-assisted deprotonation in acidic water oxidation, *Nat. Commun*. **13**, 4871 (2022).
38. Zhang, X.-X. et al. Quick evolution of edge-shared metal-oxygen octahedrons for boosting acidic water oxidation. *Nano Energy* **102**, 107680 (2022).
39. Lin, C. et al. In-situ reconstructed Ru atom array on $\alpha$-$MnO_2$ with enhanced performance for acidic water oxidation. *Nat. Catal*. **4**, 1012-1023 (2021).

40. He J., Zhou X., Xu, P. & Sun, J.-M. Regulating electron redistribution of intermetallic iridium oxide by incorporating Ru for efficient acidic water oxidation. *Adv. Energy Mater.* **11**, 2102883 (2021).
41. Liu, H. et al. Eliminating over-oxidation of ruthenium oxides by niobium for highly stable electrocatalytic oxygen evolution in acidic media. *Joule* **7**, 558-573 (2023).
42. Galyamin, D. et al. Active and durable $R_2MnRuO_7$ pyrochlores with low Ru content for acidic oxygen evolution. *Nat. Commun*. **14**, 2010 (2023).
43. Li, L. et al. Optimizing the electronics structure of ruthenium oxide by neodymium doping for enhanced acidic oxygen evolution catalysis. *Adv. Funct. Mater.* **33**, 2213304 (2023).
44. Lee, C.-S. et al. Catalyst-support interactions in $Zr_2ON_2$-supported $IrO_x$ electrocatalysts to break the trade-Off relationship between the activity and stability in the acidic oxygen evolution reaction, *Adv. Funct. Mater*. **33**, 2301557 (2023).
45. Wang, X.-J. et al. Electronic structure modulation of $RuO_2$ by $TiO_2$ enriched with oxygen vacancies to boost acidic $O_2$ evolution, *ACS Catal*. **12**, 9437-9445 (2022).
46. Huang, B. et al. Tensile-strained $RuO_2$ loaded on antimony-tin oxide by fast quenching for proton-exchange membrane water electrolyzer, *Adv. Sci*. **9**, 2201654 (2022).
47. Hao, S.-Y. et al. Dopants fixation of ruthenium for boosting acidic oxygen evolution stability and activity, *Nat. Commun*. **11**, 5368 (2020).
