# Supplementary material for: Stabilizing non-iridium active sites by non-stoichiometric oxide for acidic water oxidation at high current density
Source: Nat Commun. 2023 Nov 23;14:7644. doi: 10.1038/s41467-023-43466-x (PMC10667250; doi:10.1038/s41467-023-43466-x)
Supplement: Supplementary file 3 — Description of additional supplementary files [file 41467_2023_43466_MOESM3_ESM.pdf]

Description of Additional Supplementary Files

Supplementary Movie 1 : A movie showing the process of the binder-free Ru/TiO_x electrode working directly as an anode for water electrolysis in 0.5 M H_2SO_4 at different current densities.

Supplementary Movie 2 : A movie showing that the Ru/TiO_x electrode working directly as an anode for untreated seawater electrolysis at 100 mA cm^{-2}.
